# Association between asthma, allergic rhinitis, atopic dermatitis, and dental caries: evidence from systematic review with meta-analysis and Mendelian randomisation investigation

**DOI:** 10.7189/jogh.16.04223

**Published:** 2026-06-05

**Authors:** Qi Yu, Lei Lan, Yachun Li, Ying He, Jiaqian Hu, Kaiwen Fang, Yinhui Ji, Zhuo Chen, Ya-dong Gao, Yang Zheng

**Affiliations:** 1 Stomatology Hospital, School of Stomatology, Zhejiang University School of Medicine, Zhejiang Provincial Clinical Research Center for Oral Diseases, Zhejiang Key Laboratory of Oral Biomedical, Hangzhou, China; 2 State Key Laboratory for Diagnosis and Treatment of Infectious Diseases, National Clinical Research Center for Infectious Diseases, Collaborative Innovation Center for Diagnosis and Treatment of Infectious Diseases, The First Affiliated Hospital, Zhejiang University School of Medicine, Hangzhou, China; 3 Department of Allergy, The First Affiliated Hospital, Zhejiang University School of Medicine, Hangzhou, China

## Abstract

**Background:**

Allergic diseases and dental caries share overlapping epidemiological trends and risk factors, compromising the patients’ quality of life and increasing health care costs. We aimed to explore the associations and potential causal relationships between allergic diseases (asthma, allergic rhinitis, and atopic dermatitis) and the presence/severity of dental caries.

**Methods:**

We conducted a systematic review and meta-analysis by searching PubMed, Embase, and the Cochrane Library studies published up to 16 June 2025 reporting dental caries outcomes in relation to asthma, allergic rhinitis, or atopic dermatitis. We calculated pooled odds ratios (ORs) and standardised mean differences (SMDs) using random-effects models and utilised meta-regression to investigate heterogeneity. Then, we performed a two-sample Mendelian randomisation (MR) analysis based on genome-wide association study data to assess causal relationships, employing inverse-variance weighted (IVW) and other MR methods.

**Results:**

We included 60 studies encompassing 343 975 participants across 22 countries/regions. Asthma (OR = 1.44; 95% CI = 1.17, 1.76) and atopic dermatitis (OR = 1.09; 95% CI = 1.01, 1.17) were associated with an increased risk of dental caries, while allergic rhinitis was not (OR = 1.01; 95% CI = 0.86, 1.18). Asthma and atopic dermatitis patients had higher decayed, missing, and filled teeth for permanent dentition (DMFT) scores, with SMDs of 0.92 (95% CI = 0.32, 1.52) and 0.06 (95% CI = −0.02, 0.14). We also found higher decayed, missing, and filled surfaces for permanent dentition (DMFS) and decayed, extracted, and filled surfaces for primary dentition (defs/dfs) scores in allergic patients, while dmft/dft and the plaque index scores did not show statistically significant associations, with SMDs of 0.17 (95% CI = −0.60, 0.93) and 1.78 (95% CI = −1.47, 5.03), respectively. The MR analysis confirmed a potential causal link for asthma (IVW OR = 1.09; 95% CI = 1.02, 1.17) and a non-significant trend for atopic dermatitis (OR = 1.05; 95% CI = 0.99, 1.11).

**Conclusions:**

Our analysis showed that asthma and atopic dermatitis could be associated with an increased risk and severity of dental caries, while allergic rhinitis is not. Clinical practitioners could consider adopting an integrated prevention strategy that addresses the comorbidity between oral health and allergic diseases.

**Registration:**

PROSPERO: CRD420251103903.

Allergic diseases, which can manifest as allergic rhinitis, asthma, atopic dermatitis, and other conditions, are considered as priorities for prevention and control by the World Health Organization (WHO) [1]. Their disease burden is rising worldwide due to urbanisation and changes in living conditions [2]. Allergic rhinitis specifically was estimated to affect more than 400 million people worldwide in 2019, with prevalence rates ranging from 10–30% in adults and exceeding 40% in children [3]. Meanwhile, 260 million people are affected by asthma and 129 million by atopic dermatitis in 2021 [4]. Patients with allergic diseases often present with symptoms affecting multiple physiological systems, which complicates management [5]. Beyond their effects on respiratory and skin health, allergic diseases may influence oral health through shared epidemiologic pathways, environmental exposures, and potential side effects of treatments like reduced salivary flow from antihistamines or corticosteroids [6].

Dental caries remains the most common oral health condition, compromising patients’ quality of life and incurring significant health care costs. Epidemiological data showed this burden has remained high over the past three decades, with children exhibiting the highest disability-adjusted life year [7]. The overlapping epidemiologic trends and shared risk factors of allergic diseases and dental caries have led to the development of hypotheses on their association [8]. However, previous research has presented conflicting findings. A Korean national cross-sectional survey reported a negative correlation between dental caries and allergic rhinitis, asthma, and atopic dermatitis [9]. Conversely, two meta-analyses found a positive association between asthma and dental caries, with a pooled odds ratio of 1.06 (95% confidence interval (CI) = 1.01, 1.10) [10,11]. Mendelian randomisation (MR) studies further suggest that asthma may increase the risk of dental caries, while possibly protecting against periodontitis [12,13].

While there have been some efforts to explore the relationship between allergic diseases and dental caries, most studies have primarily focused on asthma, rather than allergic rhinitis and atopic dermatitis. Existing analyses were often restricted to specific outcomes or regionally limited data, leaving gaps in our understanding of the broader relationship with dental caries [14–16]. Moreover, allergic diseases frequently present as comorbid conditions, characterised by type 2 inflammation [17], yet their associations with dental caries and the effect sizes of these relationships remain insufficiently investigated. To address these gaps, we aimed to identify any associations and potential causal relationships between allergic diseases (asthma, allergic rhinitis, atopic dermatitis) and the presence/severity of dental caries. To this end, we conducted a systematic review and meta-analysis, synthesising evidence from population-based epidemiological studies and performing a two-sample MR analysis using genome-wide association study (GWAS) data to investigate potential causality.

## METHODS

We performed this systematic review and meta-analysis per the PRISMA guidelines and registered the protocol in PROSPERO (CRD420251103903). We searched PubMed, Embase, and the Cochrane Library using a combination of MeSH and free-text terms between the inception and 16 June 2025 (Text S1 in the **Online Supplementary Document**). We managed all retrieved records in EndNote X9 (Clarivate Analytics, Philadelphia, USA).

### Data extraction

We included cross-sectional, case-control, and cohort studies reporting on the prevalence, incident number, or rate of dental caries and at least one of three allergic diseases (allergic rhinitis, asthma, or atopic dermatitis), where diagnoses of allergic diseases used well-described definitions based on clinical symptoms and examinations, and laboratory results, and where dental caries was diagnosed through clinical examination. We excluded case reports, case series, or reviews; studies with overlapping study populations or duplicated datasets; and studies lacking original data or non-extractable data. We applied no language restrictions.

### Data extraction

Two authors (QY, LL) independently screened the titles and abstracts of all articles to exclude those that did not contain data on both allergic diseases and dental caries. They then evaluated any remaining full-text articles, cross-checking the extracted data for consistency and accuracy. Disagreements were resolved through discussion among all authors. The same two authors then extracted the following data from eligible studies using a standardised template in Microsoft Excel 2016 (Microsoft, Redmond, Washington, USA): basic study characteristics (title, first author, journal, publication year, study period, country, and design); demographic and epidemiological data (sample size, person-years of follow-up, and age range); case number of allergic diseases and dental caries; oral health measures, including the decayed, missing, and filled teeth index for primary dentition (dmft), decayed, extracted due to caries, and filled surfaces for primary dentition (defs); the decayed, missing, and filled teeth index for permanent dentition (DMFT); the decayed, missing, and filled surfaces index for permanent dentition (DMFS); and the plaque index (PI).

### Risk of bias and quality assessment

We assessed the risk of bias and quality of included studies using the Newcastle-Ottawa Scale (NOS) for cohort and case-control studies and a modified NOS for cross-sectional studies [18]. Both of these tools NOS tolls assess the three domains of selection, comparability, and outcome. The sum of the points represented the overall bias risk of each study, with 7–9 points defined as high quality for cohort and case-control studies and 8–10 points defined as high quality for cross-sectional studies [19,20]. We performed Egger’s regression-based test and visual inspection of funnel plots to assess publication bias in meta-analyses with a large number of included studies (≥10), and we used a file drawer analysis (fail-safe N calculation) to evaluate publication bias in meta-analyses with fewer studies (<10) [21]. We considered small study effects for each meta-analysis that included at least ten studies through visual inspection of funnel plots and the application of Egger’s tests [22]. Lastly, we performed a leave-one-out sensitivity analysis to assess the influence of each individual study on the final results.

### Statistical analysis

We conducted a meta-analysis incorporating both continuous (*e.g.* DMFT, DMFS) and binary caries-related outcome measures. For continuous outcomes, we calculated weighted mean differences (WMDs) with 95% CI to compare caries indices between allergic groups and non-allergic groups. We also computed standardised mean differences (SMDs) with Hedge’s g method as supplementary analyses to facilitate comparison and enhance interpretability. For studies reporting binary outcomes, we extracted raw event counts and effect sizes, *i.e.* odds ratios (ORs), with their 95% CIs. We converted the raw data of event counts into univariable effect estimates prior to meta-analysis. When both crude and multivariable-adjusted effect estimates were available, we performed a sensitivity analysis to compare the results of pooling with and without adjustment. Preference was given to the multivariable estimates to account for potential confounding variables (Text S2 in the **Online Supplementary Document**). We calculated pooled effect sizes using the DerSimonian and Laird random-effects models, and presented results as ORs with 95% CI between groups. A method based on single imputation with a prespecified value that borrows information from observed outcomes (*e.g.* the worst observed data) was used for missing data [23]. We assessed heterogeneity using the *I*^2^ statistic and explored its sources through subgroup analyses by the types of allergic diseases (asthma, allergic rhinitis, and atopic dermatitis). We further evaluated pre-specified potential key moderators, including demographic, methodological, and environmental/clinical factors, via meta-regression for outcomes with ≥10 recruited studies (Text S3 in the **Online Supplementary Document**).

### MR analysis

We obtained publicly available GWAS summary data from the IEU Open GWAS Project [24], focusing on the following GWAS IDs and sample sizes of summary-level data: asthma (ebi-a-GCST90014325, n = 408 442 samples), allergic rhinitis (finn-b-ALLERG_RHINITIS, n = 217 914), atopic dermatitis (finn-b-L12_ATOPIC, n = 205 764), and dental caries (finn-b-K11_CARIES, n = 199 565) [25] (Tables S1 and S2 in the **Online Supplementary Document****)**. We selected single-nucleotide polymorphisms (SNPs) exhibiting strong correlations with exposures as instrumental variables (IVs) (*P* < 5 × 10^−8^, clump_r^2^<0.001, and a window size of clump >10 000 kb); we assessed weak associations between SNPs and exposure by applying the F-statistic.



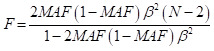



Here, *MAF* is the minor allele frequency and *β* is the estimated effect on exposure. The SNPs with an F-statistic greater than 10 were deemed reliable IVs [26,27]. We used the MR-Egger, weighted median, inverse variance weighted (IVW), simple mode, and weighted mode five MR methods to assess the influence of the exposure on the outcomes, with the IVW acting as the primary analytical method. We used the MR-Egger intercept test to assess pleiotropic associations between genetic variations and other potential confounding factors.

We used sensitivity analyses to explore the robustness of our findings and address any potential violations of the MR assumptions; a leave-one-out sensitivity test to evaluate the influence of individual SNP on the estimation of causal effects; and Cochran’s Q test to assess the heterogeneity of SNPs in both the IVW and MR-Egger methods [5].

We used ORs and 95% CI to measure the relationships between exposure and outcomes. Statistical significance was set at a *P*-value of <0.05. We performed all statistics in *R*, version 4.3.1 (R Core Team, Vienna, Austria) and conducted the MR analyses using the ‘TwoSampleMR’ and ‘MR-PRESSO’ packages [28].

## RESULTS

**Figure 1:**
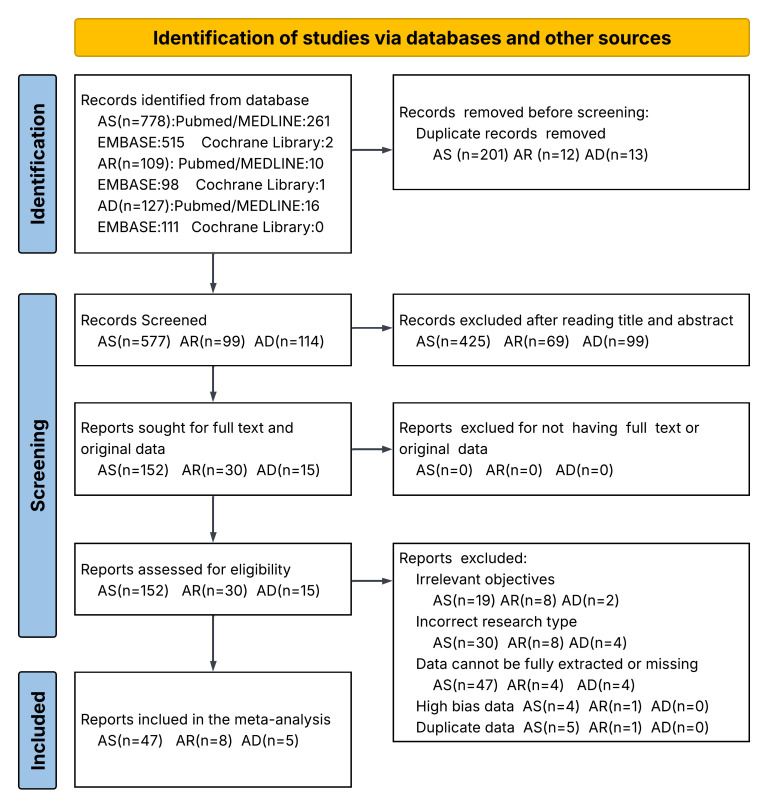
Flowchart of the study selection process.

We retrieved 1014 studies from the database, of which 226 were removed as duplicates. We excluded 593 of the remaining 790 unique records during title and abstract screening. We reviewed the full texts of the remaining 197 records and retained 60 for analysis (**Figure 1**). These 60 studies [6,9,29–82] were published between 1998 and 2025, were predominantly case-control (n = 28) and cross-sectional (n = 25) in design, had sample sizes ranging from 40 to 79 667 participants, and were performed in 22 countries/regions. The majority (n/N = 42/60) of the studies included populations that were <18 years of age (**Table 1**).

**Table 1:** Characteristics and quality assessment of included studies in the systematic review and meta-analysis

Author (year)	Country/region	Study design	Sample size	Age	Caries variables	NOS score	Quality
**AS**							
Chuang *et al*. (2018) [34]	Taiwan	Cross-sectional	9038 (1698 AS, 7340 non-AS)	2004 birth cohort	Presence of caries	9	High
Ho *et al*. (2019) [6]	Taiwan	Cohort	51 439 (7884 AS, 43 555 non-AS)	21–25	Presence of caries	9	High
Brigic *et al*. (2015) [35]	Bosnia and Herzegovina	Cross-sectional	200 (100 AS, 100 non-AS)	7–15	DMFT/PI	7	Moderate
Santos *et al*. (2012) [36]	Brazil	Cross-sectional	80 (40 AS, 40 non-AS)	10–18	DMFT/DMFS	7	Moderate
Dhinsa *et al*. (2021) [37]	India	Cross-sectional	120 (60 AS, 60 non-AS)	8–16	DMFT	7	Moderate
Shulman *et al*. (2001) [38]	USA	Cross-sectional	6938 (1129 AS, 5809 non-AS)	4–16	dft/dfs/DMFT/DMFS	9	High
Vázquez *et al*. (2011) [39]	Mexico	Cross-sectional	1160 (226 AS, 934 non-AS)	4–5	Presence of caries	7	Moderate
Shah *et al*. (2021) [40]	USA	Cross-sectional	13135 (1144 AS, 11991 non-AS)	≥18, x̄ = 46.5, SD = 15.4	Presence of caries	9	High
Chala *et al*. (2017) [41]	Morocco	Cross-sectional	200 (100 AS, 100 non-AS)	x̄ = 36.3, SD = 15.1	DMFT/PI, presence of caries	7	Moderate
Rezende *et al*. (2019) [42]	Brazil	Cross-sectional	228 (112 AS, 116 non-AS)	6–12	Presence of caries	7	Moderate
Nørrisgaard *et al*. (2023) [31]	Denmark	Cohort	589	6	Presence of caries in primary molars	9	High
Wu *et al*. (2019) [29]	Taiwan	Cohort	9190 (4601 AS, 4589 non-AS)	0–9	Presence of caries	9	High
Ramos-Ríos *et al*. (2017) [43]	Mexico	Cross-sectional	409 (28 AS, 381 non-AS)	6–12	Presence of caries	7	Moderate
Mehta *et al*. (2009) [44]	India	Case-control	160 (80 AS, 80 non-AS)	11–25	DMFT/DMFS/presence of caries	9	High
Stensson *et al*. (2011) [45]	Sweden	Case-control	40 (20 AS, 20 non-AS)	12–16	DFS/presence of caries	9	High
Botelho *et al*. (2011) [46]	Brazil	Case-control	160 (80 AS, 80 non-AS)	3–15	DMFT	9	High
Ucuncu *et al*. (2024) [47]	Turkey	Cross-sectional	62 (41 AS, 21 non-AS)	20–70, x̄ = 54.73, SD = 12.64	DMFT/DMFS	7	Moderate
Hassanpour *et al*. (2019) [48]	Iran	Case-control	140 (70 AS, 70 non-AS)	3–12	DMFT/dmft	9	High
Laurikainen *et al*. (1998) [49]	Finland	Case-control	70 (33 AS, 37 non-AS)	25–50	DMFT	9	High
Tanaka *et al*. (2008) [50]	Japan	Cross-sectional	21 792 (1663 AS, 22 0129 non-AS)	6–15	Presence of caries	7	Moderate
Mazzoleni *et al*. (2008) [51]	Italy	Case-control	60 (30 AS, 30 non-AS)	6–12	DMFT	9	High
Khalilzadeh *et al*. (2007) [52]	Iran	Case-control	91 (45 AS, 46 non-AS)	5–15	DMFT	9	High
Davidović *et al*. (2022) [53]	Bosnia and Herzegovina	Case-control	136 (68 AS, 68 non-AS)	6–16	DMFT/dmft	7	Moderate
Chellaih *et al*. (2016) [54]	India	Case-control	110 (55 AS, 55 non-AS)	6–14	DMFT	9	High
Bahrololoomi *et al*. (2018) [55]	Iran	Case-control	93 (46 AS, 47 non-AS)	6–12	dmft	7	Moderate
Alaki *et al*. (2013) [56]	Saudi Arabia	Case-control	60 (30 AS, 30 non-AS)	5–13	DMFT/dmft	9	High
Godara *et al*. (2013) [57]	India	Case-control	200 (100 AS, 100 non-AS)	10–45	DMFT/DMFS/presence of caries	9	High
Kumar *et al*. (2019) [58]	India	Case-control	140 (70 AS, 70 non-AS)	6–10	DMFT/DMFS/PI/presence of caries	9	High
Fathima *et al*. (2019) [59]	India	Case-control	200 (100 AS, 100 non-AS)	>18	DMFT	9	High
Attia *et al*. (2021) [60]	Egypt	Cohort	60 (30 AS, 30 non-AS)	6–12	DMFS/defs	9	High
Bairappan *et al*. (2020) [61]	India	Cross-sectional	100 (50 AS, 50 non-AS)	12–15	DMFT	7	Moderate
Chumpitaz-Cerrate *et al*. (2020) [62]	Peru	Case-control	184 (92 AS, 92 non-AS)	3–13	DMFT/presence of caries	9	High
Arafa *et al*. (2019) [63]	Saudi Arabia	Case-control	120 (60 AS, 60 non-AS)	4–12	DMFT	9	High
Flexeder *et al*. (2020) [64]	Germany	Cohort	730 (78 AS, 652 non-AS)	15	DMFT/presence of caries	9	High
Ersin *et al*. (2006) [65]	Turkey	Case-control	206 (106 AS, 100 non-AS)	6–19	DMFS/dfs/PI	7	Moderate
Bansal *et al*. (2022) [66]	India	Case-control	400 (200 AS, 200 non-AS)	8–15	DMFT/PI	7	Moderate
Marković *et al*. (2015) [67]	Serbia	Case-control	258 (158 AS, 100 non-AS)	2–18	DMFT/dmft/presence of caries	9	High
Stensson *et al*. (2008) [68]	Sweden	Case-control	244 (127AS, 117 non-AS)	3–6	dfs/presence of caries	7	Moderate
Stensson *et al*. (2011) [69]	Sweden	Case-control	40 (20 AS, 20 non-AS)	18–24	DFS	9	High
Ehsani *et al*. (2013) [70]	Iran	Case-control	90 (44 AS, 46 non-AS)	3–6	dmft/presence of caries	7	Moderate
Brasil-Oliveira *et al*. (2021) [71]	Brazil	Cross-sectional	90 (40 AS, 50 non-AS)	x̄ = 48.2, SD = 12.4	DMFT	7	Moderate
Katebi *et al*. (2024) [30]	Iran	Cross-sectional	1614 (538 AS, 1076 non-AS)	35–70	DMFT	9	High
Khalifa *et al*. (2014) [72]	Saudi Arabia	Case-control	120 (60 AS, 60 non-AS)	8–14	DMFT/DMFS/presence of caries	9	High
Lenander-Lumikari *et al*. (1998) [73]	Finland	Case-control	59 (26 AS, 33 non-AS)	25–50	DMFT	9	High
Świątkowska-Bury *et al*. (2022) [74]	Poland	Case-control	208 (114 AS, 94 non-AS)	3–17	DMFT/dmft/presence of caries	9	High
Kim *et al*. (2017) [9]	Korea	Cross-sectional	3703	4–15	Presence of caries	9	High
Kim *et al*. (2019) [75]	Korea	Case-control	3729 (118 AS, 3611 non-AS)	≥30	DMFT	7	Moderate
**AR**							
Kim *et al*. (2019) [75]	Korea	Case-control	3729 (486 AR, 3243 non-AR)	≥30	DMFT	7	Moderate
Bakhshaee *et al*. (2017) [76]	Iran	Cross-sectional	296 (77 AR, 219 non-AR)	5–7	DMFT	7	Moderate
Ho *et al*. (2019) [6]	Taiwan	Cross-sectional	51 439 (7884 AR, 43 555 non-AR)	21–25	Presence of caries	9	High
Segal *et al*. (2001) [77]	Israel	Cross-sectional	837 (50 AR, 762 non-AR)	x̄ = 12.57, SD = 3.29	DMFT	6	Moderate
Tanaka *et al*. (2008) [50]	Japan	Cross-sectional	21 792 (1648 AR, 20 144 non-AR)	6–15	Presence of caries	6	Moderate
Vázquez-Nava *et al*. (2008) [78]	Mexico	Cross-sectional	1160 (334 AR, 826 non-AR)	4–5	Presence of caries	6	Moderate
Vitale *et al*. (2023) [79]	Italy	Cohort	100 (50 AR ± AS, 50 non-AR)	5–14	DMFT	7	Moderate
Yılmaz *et al*. (2025) [80]	Turkey	Cohort	125 (33 AR, 30 AS, 62 non-AR)	6–12	DMFT/dmft/PI	7	Moderate
**AD**							
Tanaka *et al*. (2008) [50]	Japan	Cross-sectional	21 792 (1485 AD, 20 307 non-AD)	6–15	Presence of caries	9	High
Silverberg *et al*. (2013) [81]	US	Cross-sectional	79 667 (7188 AD, 48 415 non-AD)	0–17	Presence of caries	9	High
Smirnova *et al*. (2020) [82]	Sweden	Cross-sectional	13 151 (1264 AD, 10043 non-AD)	70+	Presence of caries	9	High
Shahin *et al*. (2025) [32]	Israel	Case-control	86 (45 AD, 41 non-AD)	18–75	Presence of caries	9	High
Park *et al*. (2021) [33]	Korea	Cross-sectional	21 606 (563 AD, 21 043 non-AD)	>18, x̄ = 46.89, SD = 14.69	DMFT	7	Moderate

AD – atopic dermatitis, AR – allergic rhinitis, AS – asthma, defs – decayed, extracted due to caries, and filled surfaces for primary dentition, dfs – decayed and filled surfaces in primary dentition for primary dentition, dft – decayed and filled primary teeth for primary dentition, dmft – decayed, missing, and filled teeth index for primary dentition, DMFS – decayed, missing, and filled surfaces for permanent dentition, DMFT – decayed, missing, and filled teeth for permanent dentition, NOS – Newcastle–Ottawa Scale, PI – plaque index, SD – standard deviation, x̄ – mean

The leave-one-out sensitivity analysis indicated that no single study significantly influenced the pooled OR or WMD for DMFT, DMFS, dmft/dft, and defs/dfs (Figures S1 and S2 in the **Online Supplementary Document**). Visual inspection of the funnel plot, Egger’s test, and fail-safe N analysis indicated potential publication bias in the DMFT and dmft/dft results, with evidence of small-study effects observed in the pooled WMD for DMFT (Figures S3 and S4 in the **Online Supplementary Document**).

**Figure 2:**
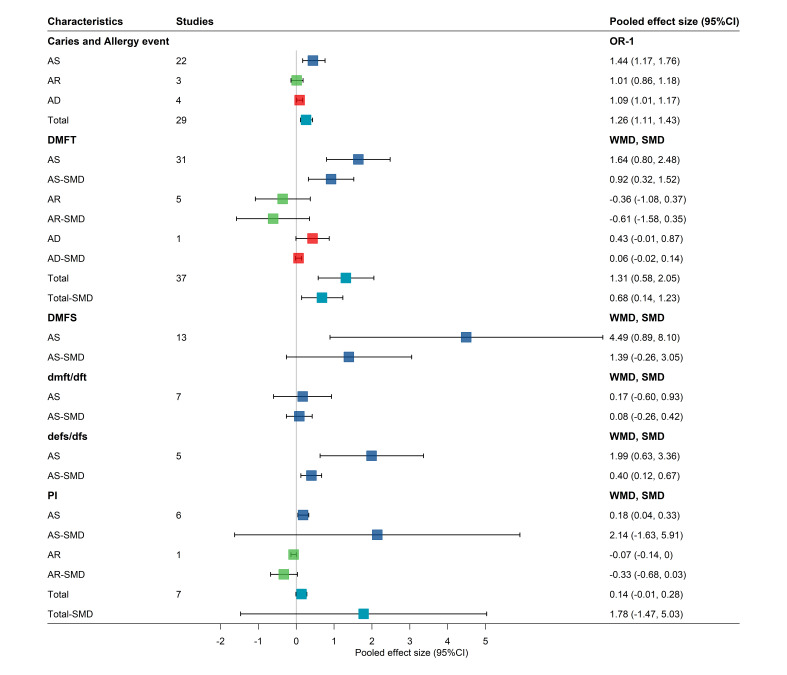
Estimation of pooled effect sizes (OR, WMD, SMD) between asthma, allergic rhinitis, atopic dermatitis, and dental caries in the meta-analysis. Squares and lines represent pooled point estimates and 95% CIs. CI – confidence interval, OR – odds ratio, WMD – weighted mean difference, SMD – standardised mean difference.

Twenty-nine records covered 26 studies reported on the association between allergic diseases and the presence of dental caries, with 22 reporting on asthma [6,9,29,31,34,39–45,50,57,58,62,64,67,68,70,72,74], 3 on allergic rhinitis [6,50,78], and 4 on atopic dermatitis [32,50,81,82]. The overall pooled OR for dental caries patients with allergic diseases vs dental caries patients without allergic diseases was 1.26 (95% CI = 1.11, 1.43). Patients with asthma and atopic dermatitis were more likely to have caries, with pooled ORs of 1.44 (95% CI = 1.17, 1.76) and 1.09 (95% CI = 1.01, 1.17), respectively, while patients with allergic rhinitis did not show such a susceptibility (OR = 1.01; 95% CI = 0.86, 1.18) (**Figure 2**; Figure S5 in the **Online Supplementary Document**). We performed meta-regression for asthma only, as it was not feasible for allergic rhinitis and atopic dermatitis due to the insufficient number of included studies. In this analysis, the risk of caries in asthma was negatively associated with socioeconomic status (OR = 0.26; 95% CI = 0.07, 0.96; *P* = 0.042), and positivity associated with asthma severity (OR = 12.42; 95% CI = 1.35, 113.90; *P* = 0.026) (Figure S6 and Table S3 in the **Online Supplementary Document**).

Thirty-seven covered 35 studies reported on the difference of DMFT between allergic patients and non-allergic patients, with 31 records from 30 studies reporting on asthma[30,35-38,41,44,46–49,51–54,56–59,61–64,66,67,71–75], 5 on allergic rhinitis [75–77,79,80], and 1 on atopic dermatitis [33]. Allergic patients had higher DMFT score, indicating a severe case of dental caries, with a WMD of 1.31 (95% CI = 0.58, 2.05) and an SMD of 0.68 (95% CI = 0.14, 1.23). The subgroup analysis showed asthma patients also had a higher DMFT score with a WMD of 1.64 (95% CI = 0.80, 2.48) and an SMD of 0.92 (95% CI = 0.32, 1.52), while allergic rhinitis patients had similar DMFT score with control groups, with a WMD of −0.36 (95% CI = −1.08, 0.37) and an SMD of −0.61 (95% CI = −1.58, 0.35). Result from the singular atopic dermatitis study revealed a higher DMFT score, with a WMD of 0.43 (95% CI = −0.01, 0.87) and an SMD of 0.06 (95% CI = −0.02, 0.14), but without statistical significance (Figure S7 in the **Online Supplementary Document**). We performed meta-regression for DMFT in asthma patients and found no significant moderating effects for any of the examined covariates (Figure S8 and Table S4 in the **Online Supplementary Document**).

Thirteen records reporting on 10 studies [36,38,44,47,57,58,60,65,69,72] focused exclusively on asthma evaluated the difference of DMFS between allergic patients and non-allergic patients. They found higher DMFS scores in asthma patients, with a WMD of 4.49 (95% CI = 0.89, 8.10) and an SMD of 1.39 (95% CI = −0.26, 3.05). Seven studies [38,48,53,55,67,70,74] reported on the dmft/dft and showed a WMD of 0.17 (95% CI = −0.60, 0.93) and SMD of 0.17 (95% CI = −0.60, 0.93). Five records covering four studies [38,60,65,68] reported on the defs/dfs and showed a higher score in asthma patients with WMD of 1.99 (95% CI = 0.63, 3.36) and SMD of 0.40 (95% CI = 0.12, 0.67) (Figure S9 in the **Online Supplementary Document**). Meta-regression analysis of DMFS in asthma patients identified socioeconomic status as a significant negative moderator (regression coefficient (*β*) = −5.46; 95% CI = −9.16, −1.76; *P*  = 0.004) and publication year as a significant positive moderator (*β*  = 0.13; 95% CI = 0.06, 0.19; *P* < 0.001), suggesting that lower socioeconomic status and more recent studies reported a progressively higher SMD (Figure S10 and Table S5 in the **Online Supplementary Document**).

Seven records covering six studies [35,41,58,65,66,80] reported on the difference of PI between allergic patients and non-allergic patients, with six reporting on asthma and one on allergic rhinitis. Allergic patients were more likely to have higher PI than non-allergic patients, with a WMD of 0.14 (95% CI = −0.01, 0.28) and an SMD of 1.78 (95% CI = −1.47, 5.03); however, this difference was not statistically significant. We also observed such a trend among asthma patients, with a WMD of 0.18 (95% CI = 0.44, 0.33) and an SMD of 2.14 (95% CI = −1.63, 5.91), and an opposite trend among allergic rhinitis patients, with a WMD of −0.07 (95% CI = −0.14, 0.00) and an SMD of −0.33 (95% CI = −0.68, 0.03) (Figure S9 in the **Online Supplementary Document**). We could not conduct a meta-regression analysis due to an insufficient number of eligible studies.

**Figure 3:**
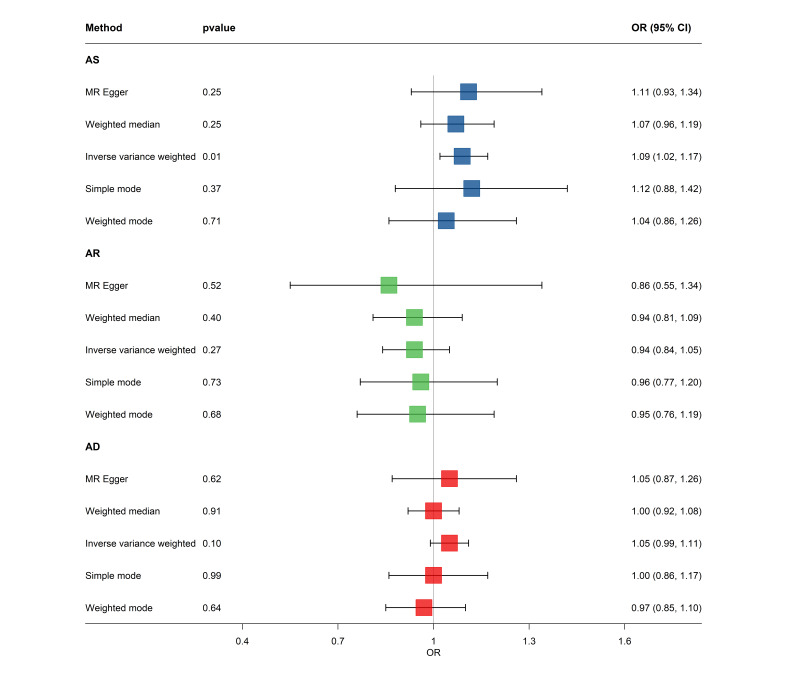
Causal relationship between asthma, allergic rhinitis, atopic dermatitis, and dental caries based on the MR.

We included 64 IVs for asthma, 10 for allergic rhinitis, and 61 for atopic dermatitis. The MR analysis showed that asthma was significantly positively associated with the increased risk of dental caries using the IVW method (OR = 1.09; 95% CI = 1.02, 1.17), while other MR methods did not reveal any statistically significant causal association. The MR analysis with five methods did not reveal association between allergic rhinitis and the risk of dental caries. The MR analysis with the IVW method revealed no significant association between atopic dermatitis and increased risk of dental caries (OR = 1.05; 95% CI = 0.99, 1.11), nor did the other MR methods (**Figure 3**). All *P*-values calculated in the MR-Egger intercept test were greater than 0.05, indicating no evidence for significant horizontal pleiotropy. The Steiger directionality test confirmed the assumed causal direction to be true. Cochran’s Q test demonstrated no evident heterogeneity under the impact of SNPs with respect to asthma (IVW: Cochran’s Q = 66.10, *P*-value for heterogeneity = 0.37), allergic rhinitis (IVW: Cochran’s Q = 3.82, *P*-value for heterogeneity = 0.92), or atopic dermatitis (IVW: Cochran’s Q = 52.03, *P*-value for heterogeneity = 0.76) (Table S6 in the **Online Supplementary Document**). Scatter plots showed a general alignment of effect directions across different estimators for allergic rhinitis and asthma, but conflicting slope directions for atopic dermatitis; the leave-one-out analysis revealed no shifts in the assessed causal effects when a single SNP was deleted (Figure S11 in the **Online Supplementary Document**).

## DISCUSSION

Our research integrated observational evidence from 60 studies comprising 343 975 participants across 22 countries/regions, with MR analyses examining the potential association between allergic diseases, including asthma, allergic rhinitis, and atopic dermatitis with dental caries. While previous meta-analyses have addressed single allergic disease and caries [8,14–16,83], our work extends their scope by providing updated quantitative synthesis comprising recently published studies incorporating multiple allergic diseases within a single analytical framework and offering complementary genetic evidence to contextualise potential causality. Our findings indicate a positive association between asthma and atopic dermatitis with increased risk of dental caries, and show no such association for allergic rhinitis. These results add to the existing body of evidence and highlight areas where further high-quality longitudinal and mechanistic studies are still needed.

The main findings of our meta-analysis and MR align with previous research that examined the association between asthma, allergic rhinitis, atopic dermatitis and dental caries. Specifically, a previous meta-analysis of 16 studies showed asthmatic children were more likely to develop caries, as determined per their higher DMFT, DMFS, dmft, and PI score [14]. Agostini *et al*. [8] reported on the estimated effects of asthma on the occurrence of caries, with pooled ORs of 1.45 (95% CI = 1.22, 1.72) and 1.52 (95% CI = 1.34, 1.73) for primary and permanent dentition, respectively. Similarly, Alavaikko *et al*. [84] reported that asthma was associated with a higher risk of dental caries, with an OR of 2.73 (95% CI = 1.61, 4.64) in primary dentition and 2.04 (95% CI = 1.44, 2.89) in permanent dentition. The heterogeneity observed in their study came from differences in geographic regions, publication years, asthma diagnostic criteria, and asthma medication use. In contrast, another meta-analysis of seven studies identified a risk effect of dental caries on asthma development, with an OR of 1.06 (95% CI = 1.01, 1.10) [11]. A two-sample bidirectional MR study revealed that asthma increased the likelihood of developing dental caries, but offered no evidence to support a causal effect of dental caries on asthma [12]. There is limited evidence from either meta-analyses or MR studies of allergic rhinitis and atopic dermatitis; only one systematic review of eight studies explored the association between allergic rhinitis and dental caries and found no support for this relationship [83].

The observational evidence from meta-analyses and genetic-driven evidence from MR studies consistently showed that asthma could increase the risk of dental caries, while allergic rhinitis could not. However, discrepancies and heterogeneity were still observed among studies of atopic dermatitis. The potential reasons for the discordant points between our meta-analysis and our MR analysis might be due to the difference in study population, inherent limitations in methodology, stratification or time-varying effects, and confounding environmental or other factors [10,85,86]. Here we synthesised five studies with a total sample size of 136 302 participants, and an *I*^2^ of 35%. However, the population varied substantially from North America, Europe, East Asia, and the Middle East, differing from the population data used in our MR (European population). Furthermore, some of our included observational studies did not fully control for confounding, wherein only a crude OR could be employed for the estimation of the pooled effect size. Thus, we made a consolidation on the robustness by assessing the IV strength, relevance, and pleiotropy, and performed a sensitivity analysis in MR; adopting adjusted effect sizes in priority, and assessing publication bias and small-study effects in meta-analysis. Although we found no statistically significant association between atopic dermatitis and dental caries (OR = 1.05; 95% CI = 0.99, 1.11), the observed marginal trend may suggest a potential link that warrants further investigation.

In our analysis, asthma and allergic rhinitis, two common respiratory allergic diseases, had different effects on the presence of dental caries. Previous evidence suggests that asthma medication might be an important determinant in the development of dental caries. Specifically, an observational study from National Health Insurance Research Database in Taiwan suggested that *β*2-agonists might contribute to caries by reducing salivary flow and increasing salivary *Streptococcus mutans* and *Lactobacillus* counts [29,87]. In contrast, the Azar cohort of 15 006 adults showed no significant association between filled teeth and the use of inhaled or oral asthma medications by multiple regression analysis [30]. Likewise, a recent prospective cohort study in Copenhagen reported that early childhood use of inhaled corticosteroids or *β*2-agonists was not linked to caries or enamel defects [31]. The discrepancy in these findings may be attributed to the improper method of inhaled technique leading to drug accumulations in the oral cavity and potentially exerting cariogenic effects [88]. Although the exact mechanisms remain unclear, the absence of association between early-life asthma medication and dental disease provides reassurance by addressing parents’ and clinicians’ concerns regarding potential drug side effects on caries [31]. In our meta-analysis, allergic rhinitis was not significantly associated with dental caries, in accordance with another recent systematic review [83]. While individual epidemiologic studies [6] indicated a potential positive relationship between allergic rhinitis and caries in our meta-analysis, most studies failed to account for confounding factors such as the use of asthma medication and socioeconomic status. Higher-quality prospective studies are needed to determine these relationship and the underlying mechanisms.

Atopic dermatitis is a skin disorder that develops due to a complex interplay between epidermal barrier dysfunction, a type 2-predominant immune response, genetic predisposition, and microbiome dysbiosis. It tends to manifest early in life as part of the ‘onset of allergic march’ [17]. Longitudinal data on the association of atopic dermatitis with dental caries are scarce. An analysis of the Growing Up in Singapore Towards healthy Outcomes study cohort found a higher prevalence of early childhood caries in atopic dermatitis patients with positive skin prick test results by the age of three years compared with children without atopic dermatitis or with negative skin prick test results. The mechanism underlying this association remains unclear; some researchers have proposed a ‘structural defect hypothesis’, whereby ectodermal defects during tissue development may contribute to both conditions [89]. A recent case-control study revealed poorer oral hygiene and dental habits among patients with atopic dermatitis, with the former potentially being further exacerbated by atopic dermatitis-related sleep disturbances [32]. Genetic polymorphisms in genes such as Dlx-3, MBL2, and TLR2, which regulate enamel formation and epidermal differentiation, could also explain the concurrent pathogenesis of skin and oral mucosal conditions [33].

This study has several limitations. First, the meta-analysis may be subject to publication bias and small-study effects, which could potentially lead to an overestimation or underestimation of the pooled effect sizes. Second, the included studies predominantly enrolled paediatric and adolescent populations, limiting the generalisability of the results to broader populations. However, as these age groups represent high-risk populations for both allergic diseases and dental caries (mainly due to their increased susceptibility to allergies and suboptimal oral hygiene practices in children and adolescents [90,91]), the results remain relevant and representative for these cohorts. Third, despite extensive meta-regression and subgroup exploration, a large proportion of heterogeneity remained unexplained in several analyses. This likely reflects unmeasured or incompletely reported factors – for example, dietary sugar intake, health care access or dental care utilisation, and diagnostic criteria for allergic diseases. Such residual heterogeneity may limit the precision and generalisability of the pooled estimates and should be considered during interpretation. Fourth, the relatively small numbers of studies on allergic rhinitis, atopic dermatitis, dmft/dft, and defs/dfs may have affected the robustness of our findings for these conditions and precluded meaningful subgroup analysis and meta-regression to explore clinical heterogeneity. Fifth, the relatively limited number of genome-wide significant SNPs available as instrumental variables for allergic rhinitis points to a need for caution when interpreting the non-significant association with dental caries. Sixth, despite our extensive sensitivity analyses, the potential for residual horizontal pleiotropy cannot be fully excluded particularly for associations of borderline significance. Thus, these results should be interpreted as suggestive and warrant further validation.

## CONCLUSIONS

As evidenced by higher DMFT and DMFS scores in our analysis, asthma and atopic dermatitis could be significantly associated with an increased risk and of dental caries. In contrast, we observed no such association for allergic rhinitis. Our findings point to the clinical rationale for an integrated prevention strategy that addresses the comorbidity between oral health and allergic diseases. We recommend that patients with specific allergic conditions be flagged for enhanced dental surveillance to mitigate their shared risk of dental caries.

## Additional material


Online Supplementary Document
