# Supplementary material for: Association between asthma, allergic rhinitis, atopic dermatitis, and dental caries: evidence from systematic review with meta-analysis and Mendelian randomisation investigation
Source: J Glob Health. 2026 Jun 5;16:04223. doi: 10.7189/jogh.16.04223 (PMC13237811; doi:10.7189/jogh.16.04223)
Supplement: Online Supplementary Document [file jogh-16-04223-s001.pdf]

Supplement to: Yu Q, Lan L, Li Y, He Y, Hu J, Fang K, Ji Y, Chen Z, Gao YD, Zheng Y. Association between asthma, allergic rhinitis, atopic dermatitis, and dental caries: evidence from systematic review with meta-analysis and Mendelian randomisation investigation. J Glob Health. 2026;16:04223.

Text S1. Method-Search strategy

1.Pubmed (MEDLINE)

1) Allergic rhinitis, caries

Search date: 2025-6-16

	Search strategy	Number of articles
#1	allergic rhinitis[MeSH Terms] OR allergic rhinitis, seasonal[MeSH Terms] OR atopic rhinitis[MeSH Terms]	24,916
#2	caries, dental[MeSH Terms] OR caries	74,552
#3	#1 AND #2	10

2) Asthma, caries

Search date: 2025-6-16

	Search strategy	Number of articles
#1	Asthma OR allergic asthma acute asthmatic attack OR wheez* OR respiratory hypersensitivity OR bronchial disorder OR hyper-responsiveness wheez* OR bronchial hyperreactivity OR airway hyperreactivity OR bronchial responsiveness OR airway responsiveness	374,553
#2	caries, dental[MeSH Terms] OR caries	74,552
#3	#1 AND #2	261

3) Atopic dermatitis, caries

Search date: 2025-6-16

	Search strategy	Number of articles
#1	(((allergic eczematous dermatitides[MeSH Terms]) OR (allergic eczematous dermatitis[MeSH Terms])) OR (atopic eczema[MeSH Terms])) OR (atopic dermatitis[MeSH Terms])	41,184
#2	caries, dental[MeSH Terms] OR caries	74,552
#3	#1 AND #2	16

2. EMBASE

1) Allergic rhinitis, caries

Search date: 2025-6-16

	Search strategy	Number of articles
#1	'allergic rhinitis' OR 'atopic rhinitis'	69,831
#2	'dental caries'/exp OR 'dental caries' OR 'caries'/exp OR 'caries'	85,870
#3	#1 AND #2	98

2) Asthma, caries

Search date: 2025-6-16

	Search strategy	Number of articles
#1	'asthma' OR 'allergic asthma' OR 'acute asthmatic attack' OR 'wheez*'	442,659
#2	'dental caries'/exp OR 'dental caries' OR 'caries'/exp OR 'caries'	85,870
#3	#1 AND #2	515

3) Atopic dermatitis, caries

Search date: 2025-6-16

	Search strategy	Number of articles
#1	'atopic dermatitis' OR 'allergic dermatitis' OR 'atopic eczema'	76,141
#2	'dental caries'/exp OR 'dental caries' OR 'caries'/exp OR 'caries'	85,870
#3	#1 AND #2	111

3. Cochrane Library

1) Allergic rhinitis, caries

Search date: 2025-6-16

	Search strategy	Number of articles
#1	MeSH descriptor: [Rhinitis, Allergic] explode all trees	3778
#2	MeSH descriptor: [Dental Caries] explode all trees	3858
#3	#1 AND #2	1
	Manual output: 1 Clinical Trial	Publication 0 matched

2) Asthma, caries

Search date: 2025-6-16

	Search strategy	Number of articles
#1	MeSH descriptor: [Asthma] explode all trees	14681
#2	MeSH descriptor: [Dental Caries] explode all trees	3858
#3	#1 AND #2	1
	Manual output : 1 Clinical Trial	Publication 2 matched

3) Atopic dermatitis, caries

Date: 2025-7-14

	Search strategy	Number of articles
#1	MeSH descriptor: [Dermatitis, Atopic] explode all trees	2764
#2	MeSH descriptor: [Dental Caries] explode all trees	3858
#3	#1 AND #2	0

Text S2. Method-Sensitivity analysis for crude versus adjusted estimation

Study	First author	Crude effects	Adjusted effects	Adjusted confounders
AS				
Allergic rhinitis, rather than asthma, might be associated with dental caries, periodontitis, and other oral diseases in adults	Ho, S. W.	1.38 (1.21-1.58)	1.03 (0.99-1.06)	Sex, socioeconomic status, urbanization, dentofacial anomalies, disease of salivary flow, DM, esophageal reflux, AS or AR
Association between asthma and dental caries in the primary dentition of Mexican children	Vázquez, E. M.	1.18 (0.82-1.70)	1.24 (0.84-1.81)	Sex, sugary products consumption antecedents and tooth brushing frequency
Association between asthma and dental caries in US (United States) adult population	Shah, P. D.	1.25 (1.10-1.42)	1.37 (1.13-1.66)	Age, Race, Gender, Smoking, BMI, Diabetes, Education and Income/Poverty Ratio
Association between untreated carious lesions and asthma in adults at Rabat University Hospital, Morocco: a cross sectional study	Chala, S.	1.42 (1.28-1.57)	1.39 (1.23-1.58)	age, sex, Occupational status, Education level,Income, Nature of previous dental attendance, Frequency of teeth bruising, Duration of teeth brushing, Age at beginning teeth brushing, Plaque index, Gingival index
Asthma medication and risk of dental diseases in children – A prospective cohort study	Nørrisgaard, P. E.	Not reported	1.09 (0.63-1.88)	plaque score, gingivitis score, and soft drinks
Dental caries and allergic disorders in Japanese children: The Ryukyus child health study	Tanaka, K.	0.97 (0.85-1.10)	0.99 (0.87-1.14)	sex, age, region of residence, number of siblings, smoking in the household, paternal and maternal history of allergy, and paternal and maternal educational level
Dental Caries Is Negatively Associated with Allergic Rhinitis, Asthma and Atopic Dermatitis in Children	Kim, S.Y.	0.57 (0.35-0.91)	0.55 (0.33-0.90)	age, sex, income level, region and number of household members
AR				

Allergic rhinitis, rather than asthma, might be associated with dental caries, periodontitis, and other oral diseases in adults	Ho, S.W.	1.75 (1.66-1.85)	1.12 (1.11-1.14)	Sex, socioeconomic status, urbanization, dentofacial anomalies, disease of salivary flow, DM, esophageal reflux, AS or AR
Dental caries and allergic disorders in Japanese children: The Ryukyus child health study	Tanaka, K.	0.88 (0.78-1.00)	0.94 (0.82-1.07)	sex, age, region of residence, number of siblings, smoking in the household, paternal and maternal history of allergy, and paternal and maternal educational level
Allergic rhinitis, feeding and oral habits, toothbrushing and socioeconomic status. Effects on development of dental caries in primary dentition	Vázquez-Nava, F.	0.84 (0.60-1.18)	0.84 (0.59-1.18)	breastfeeding duration, bottle-feeding practices, family socioeconomic status, toothbrushing frequency, sugar consumption between meals, non-nutritive sucking habits
AD				
Dental caries and allergic disorders in Japanese children: The Ryukyus child health study	Tanaka, K.	0.98 (0.85-1.14)	1.06 (0.93-1.22)	sex, age, region of residence, number of siblings, smoking in the household, paternal and maternal history of allergy, and paternal and maternal educational level
Associations of self-reported atopic dermatitis with comorbid conditions in adults: a population-based cross-sectional study	Smirnova, J.	1.25 (1.05-1.50)	1.25 (1.04-1.49)	sex, age group, smoking, education level and permanent teeth

Text S3. Method-Meta regression

Pre-specified potential key variables, including demographic (median age, proportion of males, country-level socio-demographic Index), methodological (study design, dental caries diagnostic criteria, year of publication), and environmental/clinical factors (fluoride exposure category, dentition stage, proportion with severe asthma) were evaluated via meta-regression for outcomes with ≥10 studies.

Variable	Type	Annotation
Age	Continuous	Estimated median age
Male proportion	Continuous	/
Dentition stage	Categorical	Primary, Mixed, Permanent
Socioeconomic status	Continuous	Socio-demographic Index (SDI) value corresponding to each study’s country and year of publication (sourced from the Global Burden of Disease Study, IHME) as a continuous proxy for country-level socioeconomic status
Diagnostic criteria of caries	Categorical	WHO criteria, WHO+ICDAS criteria, Other criteria
Imaging of caries	Categorical	Yes, No

Publication year	Continuous	/
Fluoride Exposure	Categorical	Three categories based on information reported in the articles and contextual national/regional background:<br>High: systemic exposure (water fluoridation) + widespread use of fluoridated toothpaste + regular professional applications<br>Moderate: routine use of fluoridated toothpaste ± occasional professional applications<br>Low: no systemic exposure and low/absent fluoridated toothpaste use
Study design	Categorical	Cross-sectional, Cohort, Case-control
Severe asthma proportion	Continuous	/

Table S1. Details of studies for mendelian randomization on asthma, allergic rhinitis, atopic dermatitis and dental caries

Data sources	GWAS ID	Year	First author	PMID	Sample size	Number of SNPs	Population
Dental caries	finn-b-K11_CARIES	2021	Kurki, M.I.	36653562	199,565	16,380,411	European
Asthma	ebi-a-GCST90014325	2021	Valette K	34103634	408,442	34,551,291	European
Atopic dermatitis	finn-b-L12_ATOPIC	2021	Kurki, M.I.	36653562	205,764	16,380,443	European
Allergic rhinitis	finn-b-ALLERG_RHINITIS	2021	Kurki, M.I.	36653562	217,914	16,380,461	European

Reference: Kurki MI, Karjalainen J, Palta P, et al. FinnGen provides genetic insights from a well-phenotyped isolated population. Nature. 2023 Jan;613(7944):508-518. doi: 10.1038/s41586-022-05473-8.

Valette K, Li Z, Bon-Baret V, Chignon A. et al. Prioritization of candidate causal genes for asthma in susceptibility loci derived from UK Biobank. Commun Biol. 2021 Jun 8;4(1):700. doi: 10.1038/s42003-021-02227-6.

Table S2. Characteristic of the genetic variants associated with allergic diseases and their effects on dental caries.

SNP	Phenotype	Effect allele	SNPs- allergic diseases			SNPs- Dental caries			R^2	F	Steiger test
			β	SE	p-value	β	SE	p-value			
rs10178845	Asthma	A	-0.0617	0.0072	8.21809E-18	-0.0032	0.0157	0.8396	0.0016	646.2645	True
rs10477741	Asthma	G	0.0743	0.0098	3.43617E-14	0.0215	0.0184	0.2427	0.0012	504.9341	True
rs10486391	Asthma	G	-0.0389	0.0067	5.1274E-09	-0.0136	0.0149	0.3601	0.0007	298.7412	True
rs10912564	Asthma	T	0.0393	0.0071	3.36817E-08	-0.0147	0.0160	0.3578	0.0007	267.7656	True
rs11042902	Asthma	T	0.0413	0.0071	6.16942E-09	-0.0198	0.0160	0.2158	0.0007	297.3406	True
rs11071559	Asthma	T	-0.0847	0.0098	4.21332E-18	-0.0172	0.0206	0.4029	0.0016	655.0344	True
rs11088309	Asthma	G	0.0605	0.0093	8.5465E-11	-0.0065	0.0216	0.7653	0.0009	365.2481	True
rs11178649	Asthma	T	-0.0429	0.0066	1.06207E-10	-0.0395	0.0155	0.0111	0.0009	363.6315	True
rs112119265	Asthma	G	-0.0778	0.0138	1.75623E-08	-0.0054	0.0221	0.8080	0.0007	277.7373	True
rs112267124	Asthma	A	0.0429	0.0077	2.55035E-08	0.0162	0.0197	0.4123	0.0007	269.6201	True
rs113981909	Asthma	A	-0.0614	0.0105	5.44702E-09	-0.0132	0.0285	0.6431	0.0008	313.6793	True
rs11567923	Asthma	CT	0.0498	0.0084	2.95206E-09	-0.0326	0.0179	0.0678	0.0008	339.6975	True
rs117552144	Asthma	T	0.0795	0.0138	8.51199E-09	-0.0185	0.0482	0.7014	0.0008	315.8626	True
rs11816044	Asthma	A	-0.0473	0.0070	1.20472E-11	-0.0267	0.0171	0.1196	0.0008	321.8567	True
rs12165508	Asthma	C	-0.0451	0.0081	2.41674E-08	-0.0143	0.0171	0.4029	0.0020	817.0473	True
rs12365699	Asthma	A	-0.0535	0.0088	1.15555E-09	-0.0187	0.0205	0.3596	0.0010	402.1670	True
rs12964116	Asthma	G	0.1076	0.0177	1.08338E-09	0.0448	0.0590	0.4475	0.0019	796.7859	True
rs13277355	Asthma	G	-0.0406	0.0073	3.09288E-08	-0.0031	0.0162	0.8480	0.0007	271.4202	True
rs1444782	Asthma	A	-0.0954	0.0066	2.13708E-47	-0.0047	0.0162	0.7723	0.0008	325.4396	True
rs148639908	Asthma	AT	-0.0707	0.0068	3.35035E-25	0.0163	0.0172	0.3437	0.0008	323.8485	True
rs1608555	Asthma	T	0.0377	0.0068	3.14193E-08	-0.0215	0.0161	0.1814	0.0014	586.9921	True
rs1684466	Asthma	A	-0.0569	0.0070	4.92366E-16	-0.0281	0.0154	0.0683	0.0007	267.5132	True

rs16903574	Asthma	G	0.0866	0.0125	5.3049E-12	-0.0047	0.0225	0.8342	0.0044	1824.9574	True
rs17454584	Asthma	G	0.0598	0.0079	3.51975E-14	0.0030	0.0157	0.8499	0.0023	936.2365	True
rs1837253	Asthma	C	0.1084	0.0075	1.48583E-47	0.0361	0.0176	0.0398	0.0007	267.8503	True
rs1870140	Asthma	G	-0.0498	0.0091	3.95975E-08	-0.0129	0.0188	0.4911	0.0015	611.8047	True
rs200491113	Asthma	GTAGA	-0.1288	0.0187	6.28128E-12	-0.0116	0.0530	0.8273	0.0014	568.7212	True
rs2296618	Asthma	G	-0.0619	0.0096	1.30432E-10	-0.0188	0.0258	0.4654	0.0011	432.8137	True
rs2412099	Asthma	A	-0.0513	0.0066	9.88519E-15	0.0001	0.0152	0.9960	0.0012	499.4732	True
rs2477923	Asthma	C	-0.0361	0.0066	3.63673E-08	-0.0054	0.0149	0.7184	0.0009	361.4384	True
rs28498223	Asthma	T	0.0497	0.0073	1.05364E-11	-0.0051	0.0163	0.7528	0.0045	1855.1853	True
rs2988277	Asthma	T	-0.0441	0.0067	3.49214E-11	0.0002	0.0170	0.9891	0.0006	264.3745	True
rs3024971	Asthma	G	-0.1122	0.0106	4.67087E-26	-0.0143	0.0361	0.6921	0.0010	420.5590	True
rs35225972	Asthma	A	0.0438	0.0070	4.41857E-10	-0.0003	0.0152	0.9840	0.0009	366.2433	True
rs35570272	Asthma	T	0.0509	0.0067	2.90541E-14	-0.0193	0.0156	0.2164	0.0013	524.7648	True
rs35621564	Asthma	G	-0.0444	0.0068	7.49953E-11	-0.0029	0.0150	0.8473	0.0006	265.3262	True
rs368981	Asthma	A	0.0562	0.0072	7.57194E-15	0.0144	0.0164	0.3812	0.0010	408.4016	True
rs3785356	Asthma	T	0.0563	0.0072	3.85208E-15	-0.0032	0.0168	0.8503	0.0009	381.5107	True
rs3827780	Asthma	A	-0.0368	0.0066	2.01683E-08	-0.0145	0.0151	0.3396	0.0024	986.0066	True
rs413214	Asthma	A	0.0469	0.0067	2.68148E-12	0.0372	0.0157	0.0177	0.0015	625.3997	True
rs4480384	Asthma	G	0.0431	0.0068	2.51675E-10	-0.0346	0.0163	0.0332	0.0037	1524.2561	True
rs4722758	Asthma	G	0.0634	0.0081	7.20698E-15	0.0111	0.0205	0.5888	0.0008	339.8856	True
rs4736639	Asthma	C	-0.0397	0.0072	4.04812E-08	-0.0218	0.0185	0.2403	0.0025	1036.1289	True
rs4795401	Asthma	G	-0.1026	0.0065	1.15551E-55	0.0066	0.0150	0.6603	0.0008	336.2837	True
rs479844	Asthma	G	0.0403	0.0066	7.70414E-10	0.0054	0.0151	0.7221	0.0012	507.8223	True
rs4842921	Asthma	A	-0.0407	0.0067	1.17001E-09	-0.0061	0.0158	0.6985	0.0009	373.5211	True
rs56375023	Asthma	A	0.1035	0.0077	3.18009E-41	0.0075	0.0169	0.6590	0.0013	532.8306	True

rs5743618	Asthma	A	-0.0664	0.0079	2.86514E-17	0.0066	0.0207	0.7493	0.0013	542.3020	True
rs5876861	Asthma	GA	-0.0374	0.0068	3.10641E-08	-0.0088	0.0167	0.5981	0.0007	274.0771	True
rs61816766	Asthma	C	0.1353	0.0187	4.50595E-13	0.1886	0.0836	0.0241	0.0010	425.9855	True
rs72823641	Asthma	A	-0.1478	0.0096	2.56014E-53	-0.0057	0.0205	0.7810	0.0009	348.4513	True
rs7423358	Asthma	C	0.0430	0.0076	1.8307E-08	0.0268	0.0164	0.1026	0.0013	525.3782	True
rs7626218	Asthma	T	-0.0414	0.0067	5.95937E-10	0.0060	0.0154	0.6944	0.0006	263.5332	True
rs76493820	Asthma	G	0.0864	0.0141	8.20882E-10	0.0108	0.0276	0.6940	0.0021	868.1602	True
rs7734635	Asthma	G	0.0768	0.0091	2.47735E-17	-0.0290	0.0188	0.1219	0.0053	2158.0971	True
rs7770794	Asthma	A	0.0416	0.0072	8.81218E-09	0.0027	0.0157	0.8653	0.0008	328.3066	True
rs78556180	Asthma	T	0.0507	0.0092	3.99482E-08	0.0186	0.0221	0.4011	0.0008	321.5417	True
rs7961712	Asthma	A	0.0565	0.0092	7.34979E-10	0.0111	0.0218	0.6088	0.0039	1588.5817	True
rs802731	Asthma	G	0.0479	0.0073	6.54243E-11	0.0064	0.0171	0.7102	0.0015	631.2189	True
rs848	Asthma	C	-0.0963	0.0085	5.79754E-30	0.0099	0.0155	0.5246	0.0007	267.6885	True
rs912131	Asthma	G	0.0581	0.0071	4.03709E-16	0.0149	0.0155	0.3372	0.0012	481.0269	True
rs9272226	Asthma	T	-0.0898	0.0095	2.3687E-21	0.0204	0.0158	0.1952	0.0052	2117.2516	True
rs9273386	Asthma	C	0.1338	0.0092	2.11488E-48	0.0464	0.0153	0.0024	0.0007	277.6584	True
rs981625	Asthma	G	0.0725	0.0132	4.16186E-08	-0.0115	0.0352	0.7434	0.0008	334.1511	True
rs10195800	Atopic dermatitis	T	0.0607	0.0106	9.16E-09	0.0021	0.0175	0.9053	0.0008	332.9926	True
rs1026788	Atopic dermatitis	C	0.0618	0.0092	1.91E-11	-0.0057	0.0150	0.7063	0.0015	629.2328	True
rs10501149	Atopic dermatitis	A	-0.0841	0.0107	3.65E-15	-0.0047	0.0173	0.7850	0.0007	288.1223	True
rs10791824	Atopic dermatitis	G	0.1030	0.0095	3.49E-27	-0.0054	0.0155	0.7257	0.0006	262.9907	True
rs1107943	Atopic dermatitis	C	0.0940	0.0170	3.31E-08	0.0356	0.0284	0.2105	0.0035	1437.1055	True
rs11156875	Atopic dermatitis	G	0.0762	0.0116	6.16E-11	-0.0097	0.0194	0.6152	0.0008	332.7415	True
rs11236813	Atopic dermatitis	C	-0.1102	0.0144	2.27E-14	-0.0367	0.0233	0.1153	0.0009	370.7498	True
rs112502960	Atopic dermatitis	A	0.0892	0.0091	1.82E-22	0.0008	0.0150	0.9557	0.0028	1132.3063	True

rs116674320	Atopic dermatitis	A	-0.0769	0.0139	2.92E-08	-0.0058	0.0225	0.7953	0.0014	575.0698	True
rs117137535	Atopic dermatitis	A	0.1573	0.0226	3.05E-12	-0.0178	0.0384	0.6433	0.0006	261.0944	True
rs117710327	Atopic dermatitis	A	-0.1114	0.0156	9.93E-13	-0.0162	0.0251	0.5189	0.0013	550.9901	True
rs11949727	Atopic dermatitis	A	-0.0745	0.0126	3.1E-09	-0.0021	0.0203	0.9178	0.0038	1543.2861	True
rs12644693	Atopic dermatitis	G	0.0580	0.0100	7.1E-09	-0.0036	0.0165	0.8295	0.0087	3573.8699	True
rs13275219	Atopic dermatitis	C	-0.0659	0.0097	1.04E-11	-0.0039	0.0159	0.8068	0.0006	260.7204	True
rs141945528	Atopic dermatitis	C	0.3356	0.0494	1.04E-11	0.0216	0.0897	0.8097	0.0054	2227.9952	True
rs142185235	Atopic dermatitis	A	0.1884	0.0185	2.89E-24	-0.0111	0.0319	0.7280	0.0038	794.0389	True
rs142841116	Atopic dermatitis	T	0.1022	0.0170	1.69E-09	0.0878	0.0282	0.0018	0.0014	297.3817	True
rs145035369	Atopic dermatitis	G	-0.1486	0.0270	0.000000036	-0.0183	0.0436	0.6743	0.0013	275.1203	True
rs1504215	Atopic dermatitis	A	-0.1035	0.0108	1.3E-21	0.0162	0.0175	0.3541	0.0039	803.1669	True
rs17371133	Atopic dermatitis	C	0.0959	0.0092	2.19E-25	0.0054	0.0151	0.7220	0.0045	931.6007	True
rs17881320	Atopic dermatitis	T	0.1019	0.0164	4.92E-10	-0.0115	0.0275	0.6747	0.0015	314.6037	True
rs182568416	Atopic dermatitis	T	0.1605	0.0262	8.6E-10	-0.0007	0.0451	0.9879	0.0014	296.0133	True
rs2041733	Atopic dermatitis	C	-0.0824	0.0092	2.41E-19	0.0031	0.0150	0.8378	0.0034	697.9980	True
rs2391683	Atopic dermatitis	G	-0.0607	0.0101	1.6E-09	-0.0333	0.0166	0.0444	0.0015	307.4494	True
rs245479	Atopic dermatitis	G	0.0607	0.0106	1.07E-08	0.0112	0.0173	0.5152	0.0014	283.9728	True
rs28377109	Atopic dermatitis	A	-0.0755	0.0117	1.03E-10	-0.0061	0.0190	0.7490	0.0018	365.1934	True
rs2893907	Atopic dermatitis	C	0.0742	0.0100	1.46E-13	0.0023	0.0163	0.8897	0.0023	478.0619	True
rs2967676	Atopic dermatitis	C	0.0730	0.0108	1.56E-11	0.0175	0.0180	0.3307	0.0018	378.6673	True
rs34290285	Atopic dermatitis	A	-0.0691	0.0112	6.4E-10	0.0078	0.0181	0.6684	0.0016	334.4011	True
rs350143	Atopic dermatitis	C	0.0684	0.0103	3.15E-11	0.0170	0.0168	0.3101	0.0019	385.6078	True
rs35570272	Atopic dermatitis	T	0.0694	0.0095	2.3E-13	-0.0193	0.0156	0.2164	0.0022	455.4766	True
rs3861950	Atopic dermatitis	C	0.0627	0.0097	9.23E-11	-0.0173	0.0160	0.2796	0.0017	352.5048	True
rs4821563	Atopic dermatitis	C	0.0643	0.0104	7.17E-10	0.0148	0.0169	0.3811	0.0016	330.4289	True

rs4851008	Atopic dermatitis	C	-0.1310	0.0114	8.99E-31	0.0091	0.0189	0.6326	0.0053	1093.9962	True
rs501764	Atopic dermatitis	T	-0.0757	0.0120	2.4E-10	-0.0101	0.0198	0.6100	0.0016	334.3946	True
rs56094005	Atopic dermatitis	G	-0.1701	0.0228	7.66E-14	-0.0187	0.0356	0.5983	0.0026	528.6443	True
rs56121811	Atopic dermatitis	T	0.0582	0.0093	4.51E-10	0.0090	0.0153	0.5553	0.0016	332.4933	True
rs6062490	Atopic dermatitis	C	0.1169	0.0118	5.28E-23	-0.0050	0.0190	0.7910	0.0042	869.9665	True
rs61816766	Atopic dermatitis	C	0.3497	0.0478	2.56E-13	0.1886	0.0836	0.0241	0.0019	388.4348	True
rs61839660	Atopic dermatitis	T	0.1443	0.0224	1.16E-10	0.0565	0.0374	0.1310	0.0016	334.2271	True
rs62089050	Atopic dermatitis	T	0.0832	0.0148	1.82E-08	0.0568	0.0249	0.0226	0.0013	258.3703	True
rs62160676	Atopic dermatitis	C	0.0550	0.0098	1.91E-08	-0.0123	0.0161	0.4450	0.0014	291.6039	True
rs6796	Atopic dermatitis	C	-0.0571	0.0098	6.42E-09	-0.0194	0.0161	0.2265	0.0014	293.2999	True
rs6852559	Atopic dermatitis	A	0.0710	0.0130	4.99E-08	0.0440	0.0216	0.0412	0.0012	248.7623	True
rs6996614	Atopic dermatitis	A	0.0760	0.0092	1.76E-16	0.0095	0.0151	0.5287	0.0028	585.2068	True
rs73018933	Atopic dermatitis	A	-0.0761	0.0107	1.02E-12	-0.0162	0.0173	0.3480	0.0022	444.6174	True
rs73068668	Atopic dermatitis	A	-0.0925	0.0169	4.56E-08	-0.0303	0.0271	0.2639	0.0013	265.9948	True
rs73222673	Atopic dermatitis	A	0.0778	0.0136	9.88E-09	0.0031	0.0225	0.8902	0.0013	275.4792	True
rs73231956	Atopic dermatitis	C	-0.0723	0.0131	3.89E-08	0.0076	0.0212	0.7190	0.0013	266.5610	True
rs76281049	Atopic dermatitis	T	-0.1482	0.0253	4.62E-09	0.0380	0.0400	0.3421	0.0015	313.1695	True
rs7688384	Atopic dermatitis	T	-0.0716	0.0099	5.03E-13	0.0029	0.0161	0.8564	0.0022	453.4457	True
rs7705653	Atopic dermatitis	G	0.0698	0.0114	1.02E-09	-0.0292	0.0190	0.1245	0.0015	311.8122	True
rs7931483	Atopic dermatitis	A	0.1200	0.0092	4.85E-39	-0.0030	0.0151	0.8424	0.0070	1449.9132	True
rs847	Atopic dermatitis	C	-0.1154	0.0094	1.31E-34	0.0114	0.0156	0.4652	0.0062	1278.2953	True
rs911263	Atopic dermatitis	T	-0.0602	0.0098	7.06E-10	0.0095	0.0161	0.5548	0.0015	319.0097	True
rs9265981	Atopic dermatitis	C	0.0679	0.0103	5.04E-11	0.0111	0.0170	0.5125	0.0018	364.3569	True
rs9310555	Atopic dermatitis	A	-0.0559	0.0091	8.82E-10	0.0002	0.0149	0.9870	0.0043	885.4128	True
rs943451	Atopic dermatitis	C	-0.0710	0.0099	7.51E-13	0.0049	0.0163	0.7632	0.0015	319.0316	True

rs964293	Atopic dermatitis	A	-0.0596	0.0098	1.23E-09	-0.0061	0.0160	0.7040	0.0021	437.2005	True
rs9859579	Atopic dermatitis	A	-0.1134	0.0147	1.26E-14	0.0008	0.0237	0.9718	0.0016	321.2054	True
rs987106	Atopic dermatitis	T	-0.0670	0.0094	8.18E-13	-0.0031	0.0154	0.8386	0.0026	531.1736	True
rs10786678	Allergic rhinitis	T	0.0767	0.0125	7.46879E-10	-0.0053	0.0151	0.7285	0.0021	433.4228	True
rs11465723	Allergic rhinitis	A	-0.1077	0.0170	2.16028E-10	-0.0009	0.0202	0.9663	0.0032	697.2710	True
rs11751024	Allergic rhinitis	A	-0.0837	0.0126	3.74283E-11	-0.0086	0.0152	0.5702	0.0034	733.8582	True
rs12233670	Allergic rhinitis	T	-0.1522	0.0193	3.26362E-15	0.0096	0.0223	0.6670	0.0052	1132.1364	True
rs12349858	Allergic rhinitis	T	0.0980	0.0170	7.9805E-09	0.0026	0.0210	0.9002	0.0024	529.2837	True
rs1438673	Allergic rhinitis	T	-0.1065	0.0124	6.72667E-18	0.0281	0.0150	0.0607	0.0056	1229.4221	True
rs76715626	Allergic rhinitis	C	-0.1781	0.0272	5.98549E-11	0.0117	0.0317	0.7130	0.0036	777.2739	True
rs7936312	Allergic rhinitis	T	0.0919	0.0124	1.57217E-13	-0.0045	0.0151	0.7666	0.0041	896.4994	True
rs847	Allergic rhinitis	C	-0.0770	0.0128	1.7386E-09	0.0114	0.0156	0.4652	0.0027	600.3434	True
rs9442514	Allergic rhinitis	T	-0.0772	0.0139	2.58541E-08	-0.0062	0.0166	0.7089	0.0024	525.9321	True

SNP, single nucleotide polymorphism; EA, effect allele; SE, standard error.

R^2 were calculated using the following formula: $2 \times MAF \times (1-MAF) \times Beta^2$, where MAF is the minor allele frequency , Beta is the estimated effect on exposure.

F were calculated using the following formula: $R^2(N-2)/(1- R^2)$, where R^2 is the proportion of variance in exposure explained by each instrument.

Figure S1. Leave-one-out forest plot of the association between allergic diseases and the presence of dental caries

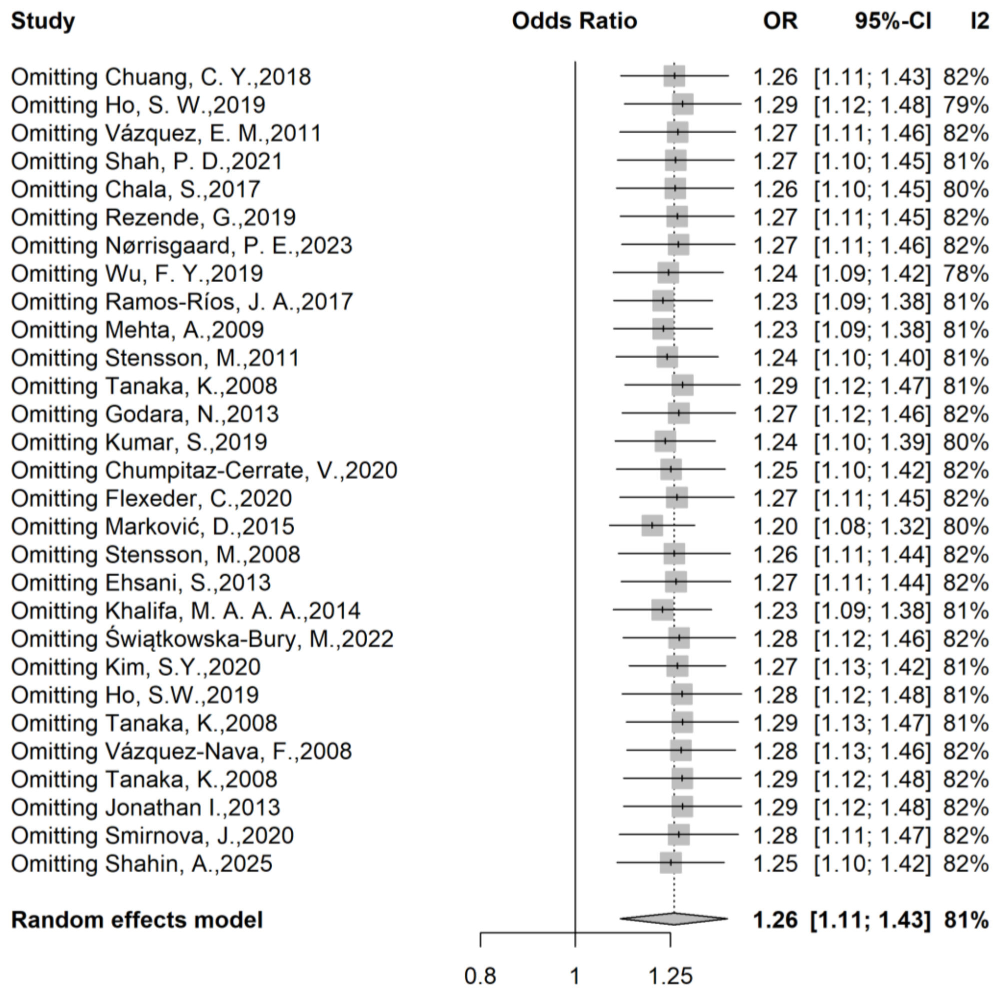

Leave-one-out sensitivity analysis demonstrated that the pooled effect size remained robust across all iterations, with effect estimates ranging from 1.20 (95% CI 1.08—1.32) to 1.29 (95% CI 1.12—1.48).

Figure S2. Leave-one-out forest plot of DMFT

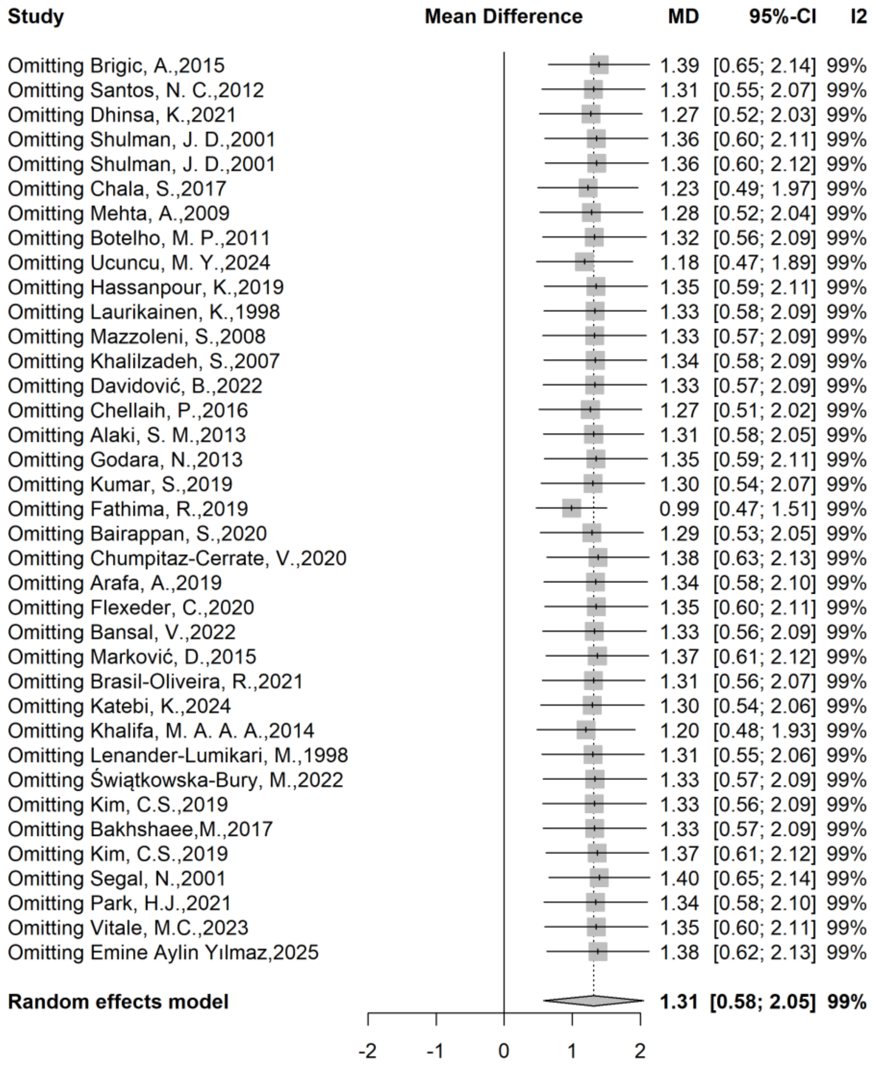

Leave-one-out sensitivity analysis demonstrated that the pooled WMD remained robust across all iterations, with effect estimates ranging from 0.99 (95% CI 0.47—1.51) to 1.40 (95% CI 0.65—2.14).

Figure S3. Funnel plot of the studies showing the association between allergic diseases and the presence of dental caries

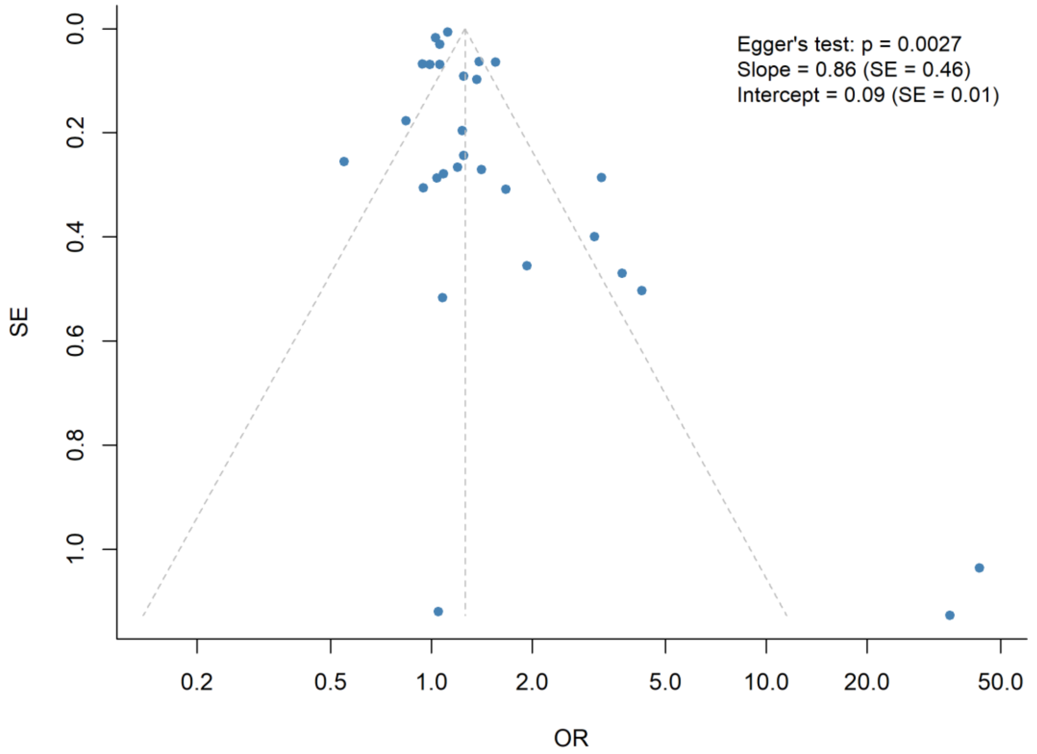

The linear regression test for funnel plot asymmetry (Egger's test) showed no statistically significant evidence of publication bias (t= 1.89, p = 0.070). The intercept of 0.093 (SE = 0.015) suggests minimal small-study effects, though the positive direction indicates a potential tendency for smaller studies to report marginally larger effects.

Figure S4. Funnel plot before (left) and after (right) the trim-and-fill analysis of the studies showing DMFT

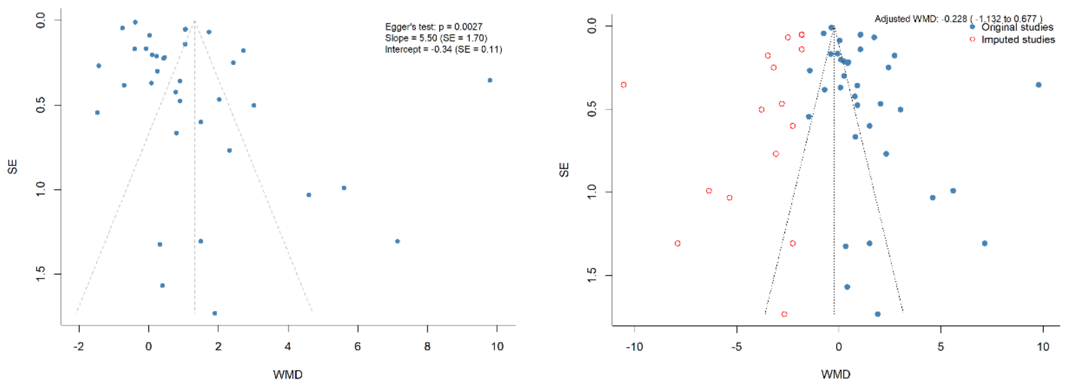

Egger's test for funnel plot asymmetry revealed significant publication bias or small-study effects ($t = 3.24$, $df = 34$, $p = 0.0027$), with a positive bias coefficient (5.50, SE = 1.70) indicating that smaller studies tend to report larger effect sizes. The trim-and-fill method was applied to adjust for this asymmetry

Figure S5. Forest plot of allergic diseases and dental caries presence

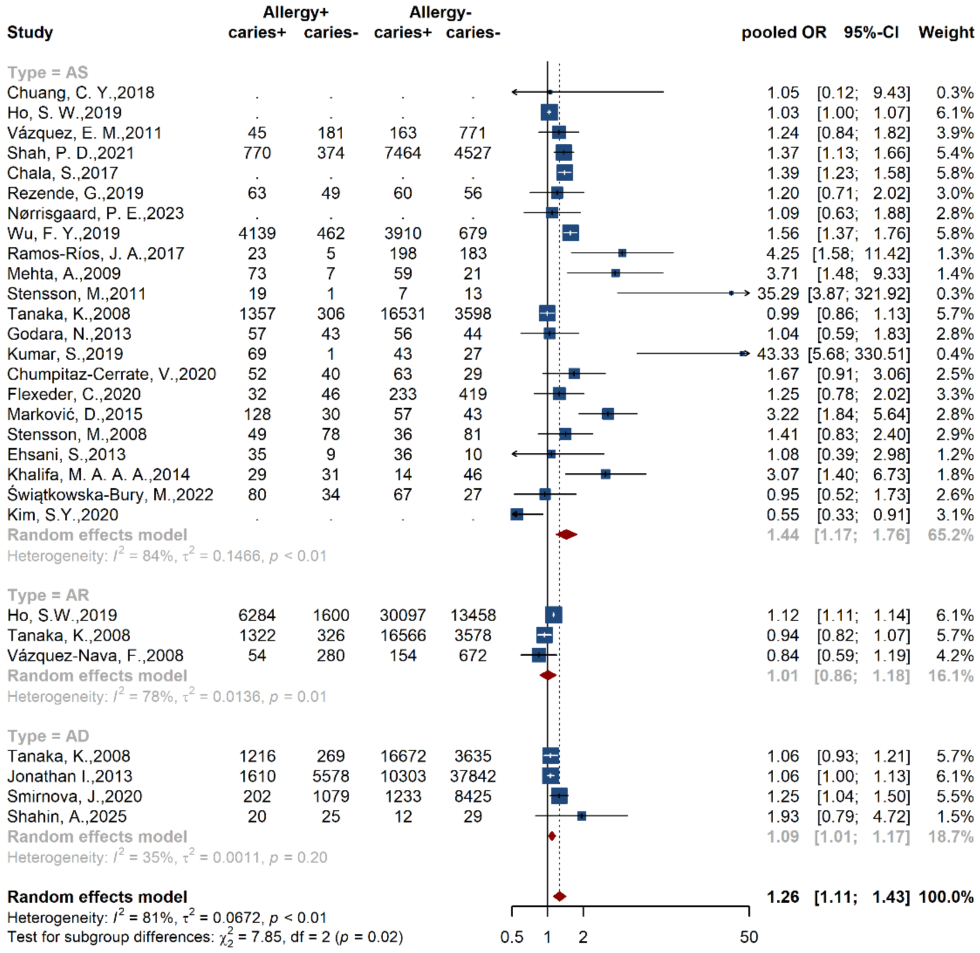

Table S3. Meta-regression results for studies reporting the presence of caries in asthma

Variable	Type/unit	Study number	OR (95% CI)	P value	I^2 (%)
Median Age	Per year-old	22	0.998 (0.986-1.011)	0.767	84.7
Male proportion	Per 1%	20	2.086 (0.389-11.19)	0.391	85.5
SES	Per 1 unit	22	0.257 (0.069-0.955)	**0.042**	79.2
Publication year	Per year	22	0.994 (0.966-1.022)	0.664	84.2
Severe asthma proportion	Per 1%	9	12.42 (1.354-113.9)	**0.026**	71.1
Dentition stage	Primary	22	Ref		82.2
	Mixed	22	1.122 (0.663-1.899)	0.667	
	Permanent	22	1.085 (0.614-1.917)	0.778	
Caries diagnostics criteria	Others	22	Ref		84.4
	WHO	22	1.021 (0.696-1.497)	0.916	
	WHO+ICDAS	22	0.764 (0.441-1.325)	0.338	
Imaging in caries diagnosis	Yes vs. No	22	1.420 (0.696-2.899)	0.335	84.5
Fluoride exposure	Low	9	Ref		
	Moderate	9	0.964 (0.305-3.046)	0.950	75.9
	High	9	0.727 (0.245-2.161)	0.567	
Study design	Case-control	22	Ref		82.4
	Cohort	22	0.650 (0.416-1.017)	0.059	
	Cross-sectional	22	0.720 (0.470-1.101)	0.129	

Socioeconomic status (SES) and disease severity significantly moderated the association between asthma and dental caries. Each one-unit increase in SES (presented as SDI) corresponded to a reduced OR of 0.257, indicating a protective effect of higher socioeconomic status. Patients with severe asthma showed a significantly higher caries risk. Study design exhibited a borderline significant moderating trend, with cohort studies demonstrating a lower effect size than case-control studies. Other covariates, including age, gender, and publication year, showed no significant moderating effects. The analysis indicated moderate-to-high heterogeneity (I^2: 71.1%–85.5%), suggesting the presence of potential unmeasured influencing factors.

Figure S6. Bubble plots and forest plot of variables moderating the association between asthma and dental caries

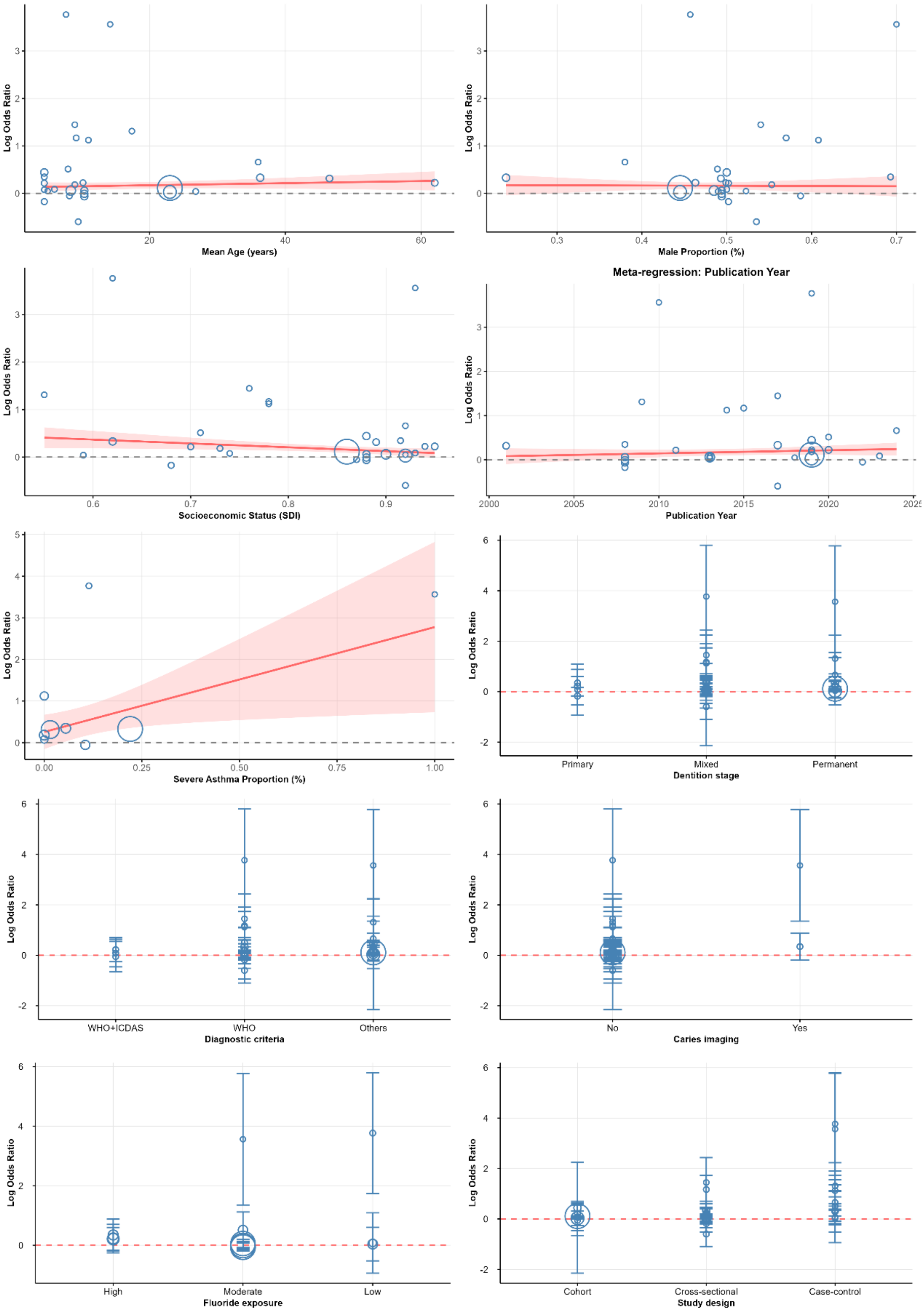

Figure S7. Forest plot of the mean difference and standardized mean difference of DMFT

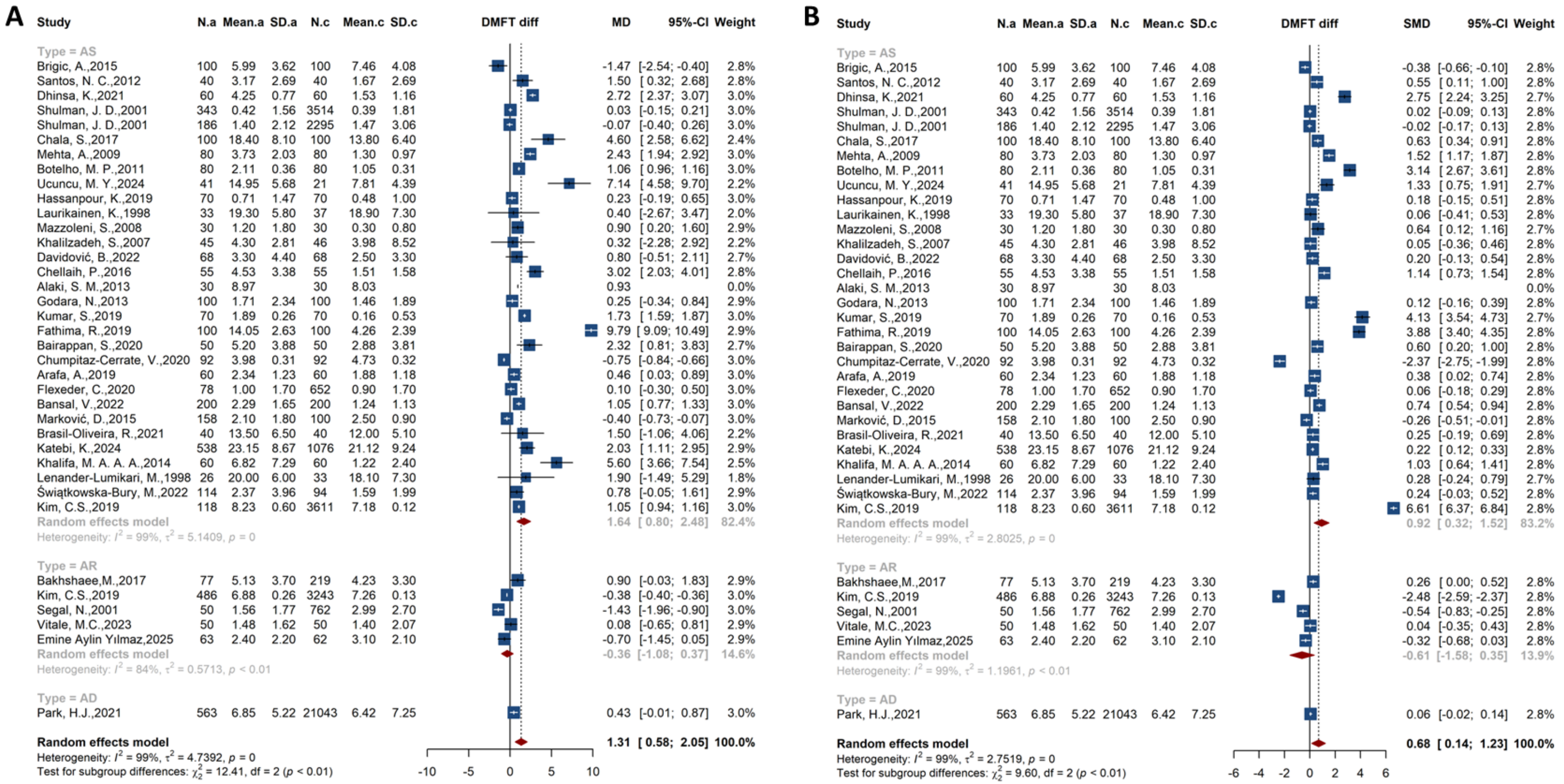

A. Result of mean difference (MD) of DMFT; B. Result of SMD of DMFT.

Table S4. Meta-regression results for studies reporting the DMFT in asthma

Variable	Type/unit	Study number	β (95% CI)	P value	I² (%)
Median Age	Per year-old	30	0.034 (-0.004, 0.723)	0.079	99.15%
Male proportion	Per 1%	24	-2.899 (-8.134, 2.337)	0.278	99.36%
SES	Per 1 unit	30	-1.622 (-6.675, 3.432)	0.529	99.23%
Publication year	Per year	30	0.038 (-0.040, 0.116)	0.338	99.20%
Severe asthma proportion	Per 1%	12	-0.311 (-2.534, 1.912)	0.784	95.31%
Dentition stage	Mixed	30	Ref		
	Permanent	30	0.631 (-0.511, 1.774)	0.279	99.17%
Caries diagnostics criteria	Others	30	Ref		99.25%
	WHO	30	0.462 (-1.432, 2.355)	0.633	
	WHO+ICDAS	30	-0.413 (-3.236, 2.411)	0.775	
Imaging in caries diagnosis	Yes vs. No	30	All not use		
Fluoride exposure	Low	10	Ref		98.34%
	Moderate	10	-1.832(-4.164, 0.499)	0.120	
	High	10	-0.177 (-2.508, 2.155)	0.882	
Study design	Case-control	30	Ref		99.20%
	Cohort	30	-0.420 (-2.844, 2.004)	0.734	
	Cross-sectional	30	0.630 (-0.608, 1.869)	0.319	

None of the potential variables reached statistical significance (all $p > 0.05$). Age exhibited a marginally significant moderating trend ($\beta = 0.034$, $p = 0.079$), suggesting that each one-year increase in age may be associated with an increase of 0.034 standard deviations in the DMFT difference, though this effect is small and supported by limited statistical evidence. All analyses exhibited extremely high heterogeneity (I^2: 95.31%–99.36%), indicating the presence of other important unmeasured influencing factors.

Figure S8. Bubble plots and forest plot of variables moderating DMFT

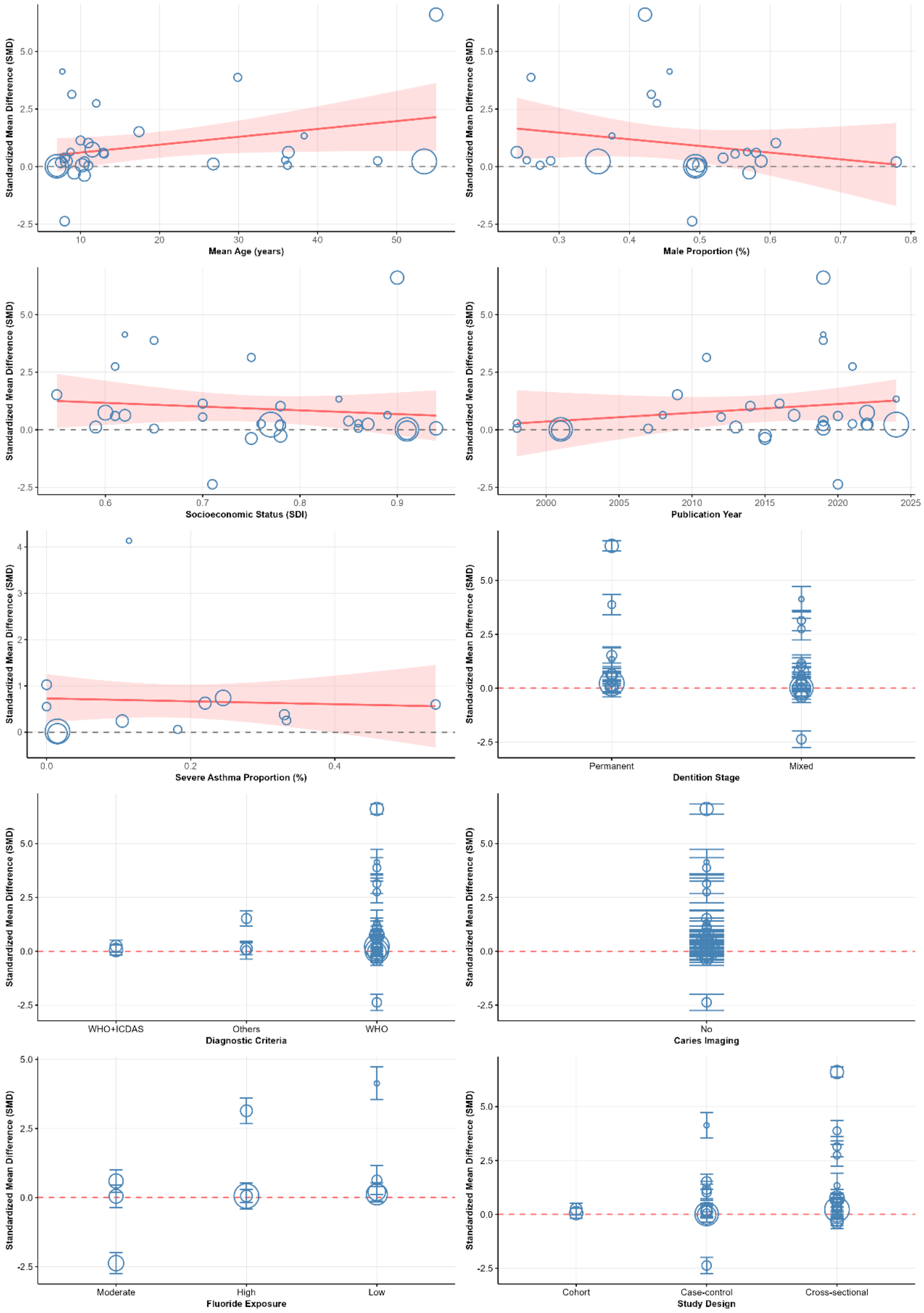

Figure S9. Forest plot of the mean difference and standardized mean difference of other results

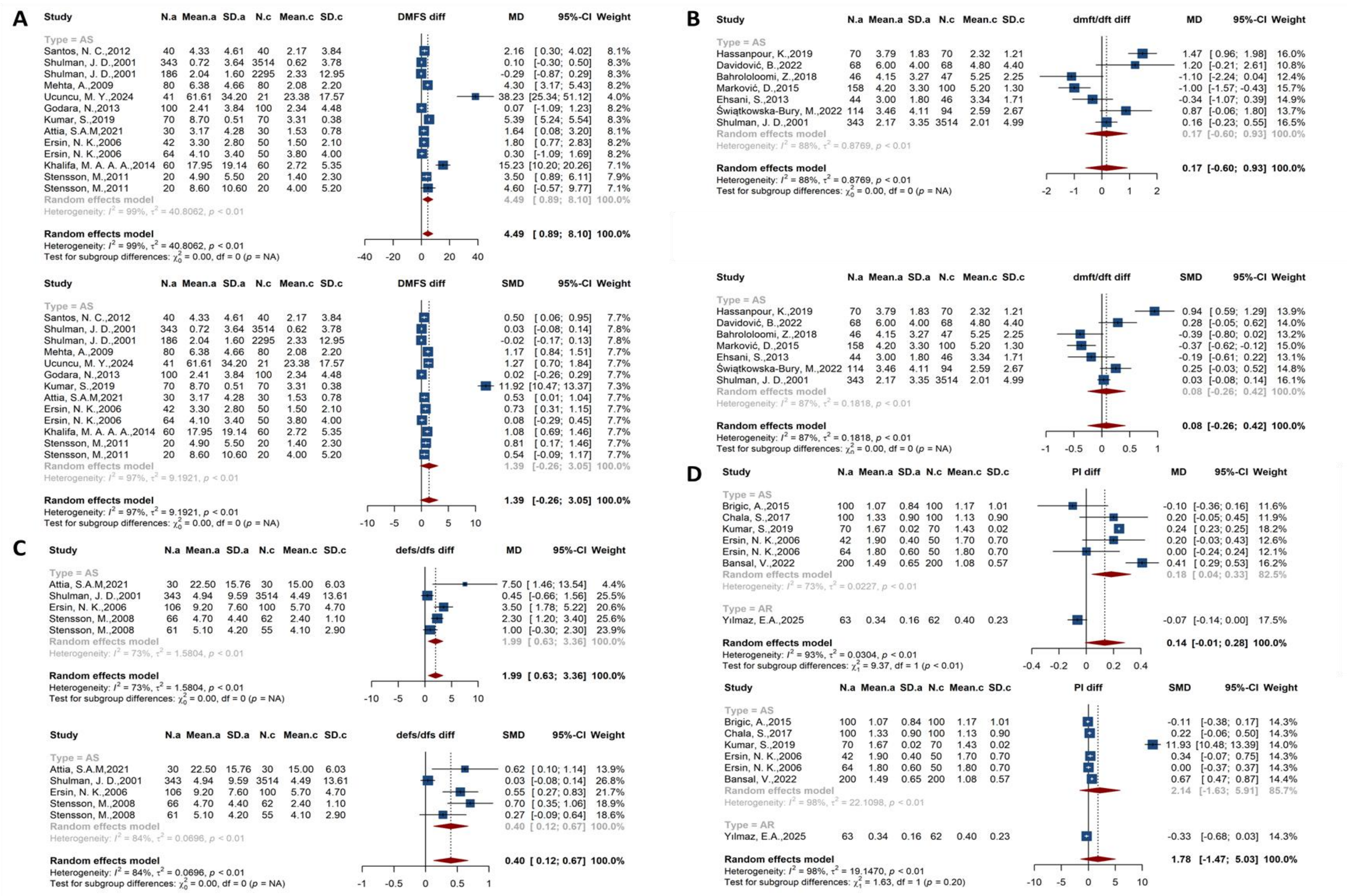

A. Result of mean difference (MD) and SMD of DMFS;

B. Result of mean difference (MD) and SMD of dmft/dft;

C. Result of mean difference (MD) and SMD of defs/dfs;

D. Result of mean difference (MD) and SMD of PI;

Table S5. Meta-regression results for studies reporting the DMFS in asthma

Variable	Type/unit	Study number	β (95% CI)	P value	I^2 (%)
Median Age	Per year-old	13	-0.031 (-0.086, 0.023)	0.263	96.67%
Male proportion	Per 1%	12	-2.791 (-7.467, 1.885)	0.242	96.81%
SES	Per 1 unit	13	-5.462 (-9.161, -1.764)	**0.004**	96.36%
Publication year	Per year	13	0.126 (0.061, 0.191)	**<0.001**	95.93%
Severe asthma proportion	Per 1%	9	0.283 (-1.695, 2.261)	**0.779**	97.61%
Dentition stage	Mixed	13	Ref		
	Permanent		-0.544 (-1.523, 0.435)	0.276	96.67%
Caries diagnostics criteria	Others	13	Ref		96.76%
	WHO		0.571 (-0.564, 1.705)	0.324	
	WHO+ICDAS		No		
Imaging in caries diagnosis	Yes vs. No	13	-0.472 (-1.784, 0.841)	0.481	96.81%
Fluoride exposure	Low	6	Ref		98.82%
	Moderate		-5.192(-9.785, -0.599)	0.027	
	High		-5.192 (-11.341, 0.956)	0.098	
Study design	Case-control	13	Ref		97.05%
	Cohort		-0.879 (-2.722, 0.964)	0.350	
	Cross-sectional		-0.769 (-1.828, 0.291)	0.155	

socioeconomic status as a significant negative moderator, and publication year as a significant positive moderator, suggesting that lower socioeconomic status and more recent studies reported a progressively higher SMD. All analyses exhibited high heterogeneity (I^2), indicating the presence of other important unmeasured influencing factors.

Figure S10. Bubble plots and forest plot of variables moderating DMFS

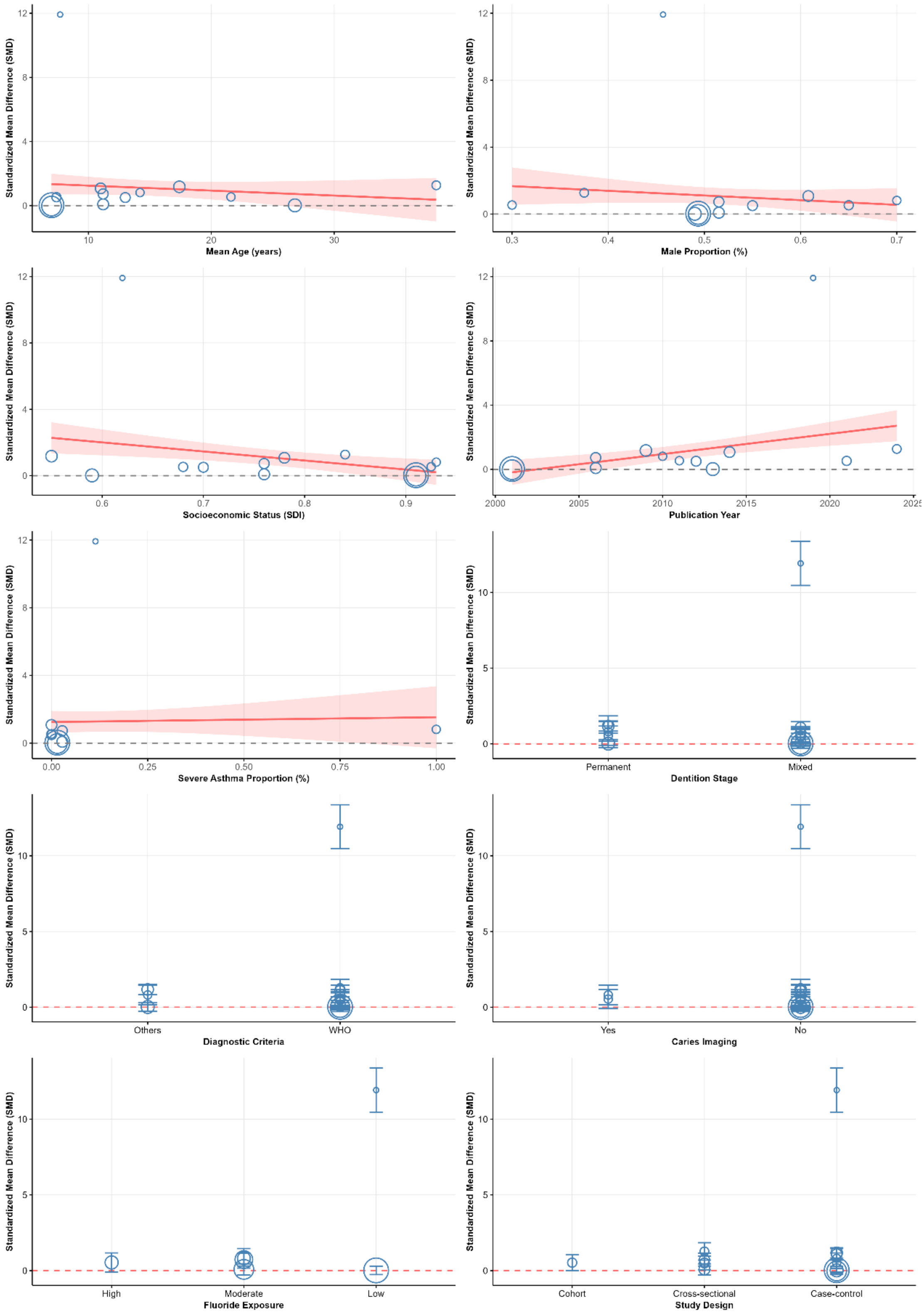

Table S6. Verification of the associations between asthma, allergic rhinitis, atopic dermatitis and dental caries

Phenotype	Method	SNPs(N)	OR	95%CI	MR p-Value	Heterogeneity Q /p-value	MR-PRESSO pleiotropy intercept /p-value
Asthma	MR Egger	64	1.11	(0.93,1.34)	0.25	66.05/0.3387	-0.0012/0.8371
	Weighted median	64	1.07	(0.96,1.19)	0.25		
	Inverse variance weighted	64	1.09	(1.02,1.17)	0.01	0.37/0.3704	
	Simple mode	64	1.12	(0.88,1.42)	0.37		
	Weighted mode	64	1.04	(0.86,1.26)	0.71		
Atopic dermatitis	MR Egger	61	1.05	(0.87,1.26)	0.62	52.03/0.7281	-0.0002/0.9835
	Weighted median	61	1.00	(0.92,1.08)	0.91		
	Inverse variance weighted	61	1.05	(0.99,1.11)	0.11	52.03/0.7584	
	Simple mode	61	1.00	(0.86,1.17)	0.99		
	Weighted mode	61	0.97	(0.85,1.1)	0.64		
Allergic rhinitis	MR Egger	10	0.86	(0.55,1.34)	0.52	3.65/0.8874	0.0093/0.6896
	Weighted median	10	0.94	(0.81,1.09)	0.40		
	Inverse variance weighted	10	0.94	(0.84,1.05)	0.27	3.82/0.9229	
	Simple mode	10	0.96	(0.77,1.2)	0.73		
	Weighted mode	10	0.95	(0.76,1.19)	0.68		

SNP, single nucleotide polymorphism; OR, odds ratio; CI, confidence interval; MR, Mendelian randomization.

Figure S11. The scatter plots about SNP effects and leave-one-out sensitivity test on asthma, allergic rhinitis, atopic dermatitis and dental caries

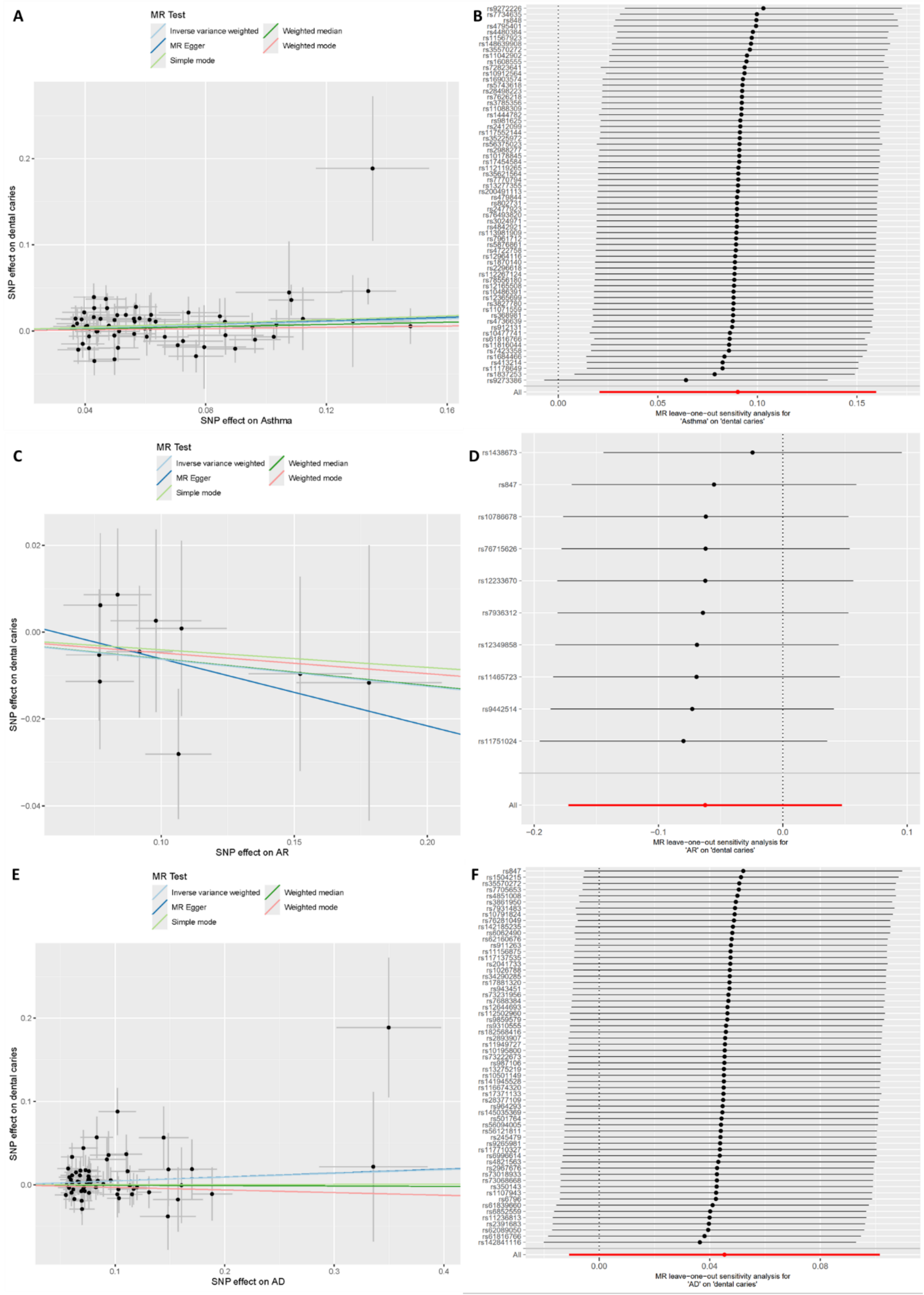

A. scatter plots about SNP effects on asthma; B. leave-one-out sensitivity analysis on asthma;

C. scatter plots about SNP effects on AR; D. leave-one-out sensitivity analysis on AR;

E. scatter plots about SNP effects on AD; F. leave-one-out sensitivity analysis on AD.
